# Electroplating Carbon Nano‐Onion on Copper for Dendrite‐Free and Anode‐Free Zinc‐Ion Batteries

**DOI:** 10.1002/advs.202510617

**Published:** 2025-08-13

**Authors:** Yufan Zhang, Ramu Banavath, Shegufta Upama, Sayyam Deshpande, Huaixuan Cao, Brian R. Stepp, Navid Attarzadeh, Stephnie Peat, Joseph V. Kosmoski, Evan C. Johnson, Micah J. Green

**Affiliations:** ^1^ Artie McFerrin Department of Chemical Engineering Texas A&M University College Station TX 77843 USA; ^2^ Department of Material Science and Engineering Texas A&M University College Station TX 77843 USA; ^3^ Nabors Energy Transition Solutions LLC Houston TX 77067 USA

**Keywords:** anode‐free batteries, carbon nano‐onions, current collector, dendrite‐free, electroplating, zinc‐ion batteries

## Abstract

Zinc‐ion batteries (ZIBs) have emerged as a promising and safer alternative to traditional lithium‐ion batteries (LIBs) due to their cost‐effectiveness, material abundance, and the use of non‐flammable aqueous electrolytes; however, the widespread adoption of ZIBs is still hindered by challenges such as dendrite formation, hydrogen evolution, and electrode passivation. Herein, the electroplating of carbon nano‐onions (CNOs) and Cu is demonstrated to form a CNO‐embedded‐Cu coating onto Cu foil for use as Zn anode current collectors, which facilitates high zinc plating/stripping efficiency (99.89%) over 1400 cycles. This CNO‐embedded‐Cu coating ensures high conductivity of the substrate surface and stabilizes Zn deposition by embedded CNOs. This CNO@Cu film achieves long‐term cycling with an 85.5% depth of discharge over 176 h, outperforming traditional Cu (102 h). Further, a ZIB fabricated with a CNO@Cu current collector in an anode‐free configuration paired with a zincified NH_4_V_4_O_10_ cathode demonstrates a high volumetric energy density of 194.8 Wh L^−1^. CNO@Cu offers a low‐cost, scalable, and high‐performance solution for industrial anode‐free ZIBs, and this work sets the foundation for future improvements in ZIBs by utilizing cost‐effective, industrial‐grade nanomaterials.

## Graphical abstract

Zinc‐ion batteries are emerging as safe and cost‐effective alternatives to lithium‐ion batteries, but suffer from dendrite formation and unstable anodes. An electroplating approach for preparing a carbon nano‐onion (CNO)‐embedded‐Cu layer on Cu foil is presented, which enables highly reversible zinc plating/stripping (99.89% efficiency over 1400 cycles) and delivers a high volumetric energy density of 194.8 Wh L^−1^ in an anode‐free configuration.

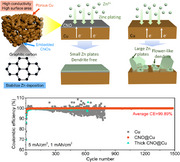

## 1 Introduction

Energy storage technology has become an essential pillar supporting the advancement of renewable energy systems, electric vehicles, and various portable electronics.^[^ ^1^, ^2^, ^3^ ^]^ Currently, lithium‐ion batteries (LIBs) dominate the market due to their high energy density, long cycle life, and being a relatively mature technology.^[^ ^4^, ^5^, ^6^ ^]^ However, the limitations associated with LIBs have prompted researchers to explore alternative battery chemistries that offer improved sustainability, cost‐efficiency, and safety.^[^ ^7^ ^]^ As an alternative to LIBs, aqueous zinc‐ion batteries (ZIBs) have emerged as one of the most promising candidates for next‐generation energy storage systems.^[^ ^8^, ^9^, ^10^ ^]^ The appeal of ZIBs lies in the abundance and low cost of zinc, as well as the inherent safety of aqueous electrolytes. The use of aqueous electrolytes in ZIBs not only eliminates the risk of fire and explosion but also simplifies battery manufacturing and recycling processes. Zinc metal offers several advantages that make it a suitable candidate for energy storage.^[^ ^11^, ^12^ ^]^ For example, zinc possesses a high theoretical capacity (819 mAh g^−1^ or 5855 mAh cm^−3^) and a low redox potential (−0.76 V vs standard hydrogen electrode), which enables it to serve as both an efficient anode material and an energy‐dense storage medium.^[^ ^13^, ^14^, ^15^ ^]^ Furthermore, ZIBs exhibit rapid charge–discharge rates, making them suitable for applications requiring high power density, such as grid‐scale storage, backup power systems, and electric vehicles.^[^ ^16^, ^17^ ^]^ Given these factors, ZIBs have the potential to be a key technology in the shift toward sustainable energy.

However, despite their advantages, the commercial viability of ZIBs is hampered by several critical challenges.^[^ ^18^, ^19^, ^20^ ^]^ One of the most pressing issues is the tendency for zinc to form dendrites during repeated charge–discharge cycles. Zinc dendrites grow on the anode surface during plating, and over time, these dendrites can penetrate the separator, leading to short circuits and battery failure. This issue is exacerbated in aqueous systems, where hydrogen evolution reactions can occur at the zinc anode, competing with the zinc deposition process and further contributing to swelling in the battery, poor cycle life, and reduced Coulombic efficiency (CE). In addition, passivation of the zinc anode, where an insulating layer forms on the surface, can significantly hinder the efficiency of zinc plating and stripping. Overcoming these issues is essential for the development of commercially viable ZIBs. Various strategies have been employed to address these problems, including the modification of the ZIB anode.^[^ ^12^, ^21^, ^22^, ^23^, ^24^, ^25^, ^26^ ^]^

One promising approach is the use of carbon nanomaterials to modify the anode current collector surface, providing more favorable sites for zinc deposition and mitigating dendrite growth.^[^ ^27^, ^28^ ^]^ This modification is typically limited to simple drop‐casting of carbon nanomaterials onto the surface. Carbon nanomaterials such as graphene and carbon nanotubes (CNTs) have been extensively studied for this purpose due to their similar lattice structure to zinc metal, high electrical conductivity, and large surface area.^[^ ^27^, ^29^, ^30^ ^]^ Recent studies have further emphasized the role of carbon interfaces in regulating Zn deposition. For instance, conductive carbon nano‐architectures have been shown to promote uniform Zn nucleation,^[^ ^31^ ^]^ while hybrid Cu/carbon scaffolds have been reported to enhance Zn plating reversibility.^[^ ^32^ ^]^ By providing uniform nucleation sites for zinc plating, these materials can suppress dendrite growth and improve the overall performance of the battery. However, the cost of synthesizing high‐grade carbon nanomaterials remains prohibitively high for large‐scale industrial applications.

To overcome production limitations associated with certain nanomaterials, researchers have begun to explore more cost‐effective and mass‐produced alternatives, such as carbon nano‐onions (CNOs). In general, CNOs are a form of carbon nanomaterial comprising graphene‐like sheets assembled into multi‐layered carbon nanoparticles, which have been used as conductive additives in various applications, including batteries, supercapacitors, and electrocatalysis.^[^ ^33^, ^34^, ^35^, ^36^, ^37^, ^38^ ^]^

Previous studies have shown that deposited CNOs can enhance the electrochemical performance of zinc anodes. For example, Peng et al. demonstrated that CNOs could be used as an interfacial layer to stabilize the zinc anode, achieving a Zn plating/stripping efficiency of 99.4% on a CNO film on Cu.^[^ ^39^ ^]^ However, due to the relatively low conductivity and low surface area of these CNOs compared to graphene and CNTs, the Zn plating/stripping performance on CNO‐modified zinc anodes has remained suboptimal (99.4% CE for CNOs vs 99.9% CE for graphene^[^ ^27^ ^]^). Further improvements in the fabrication methods are necessary to fully realize the potential of CNOs in ZIBs.

Recently, we reported a combustion‐based synthesis method that does not require catalysts, enabling large‐scale production of CNO used in this study.^[^ ^35^, ^40^ ^]^ Working with this carbon nanomaterial, we have developed a novel electroplating method to prepare a thin, transparent layer of CNO‐embedded‐Cu layer onto the Cu current collector to stabilize the Zn anode, ensuring that the current collector retains its high electrical conductivity, while the CNOs promote Zn plating stability. We have successfully achieved a zinc plating/stripping efficiency of 99.89% on CNO@Cu over 1400 cycles, significantly improving upon the performance reported in previous studies. In addition, Zn foil on our CNO@Cu film exhibits long‐term cycling stability of 176 h at 85.5% depth of discharge (DOD) for symmetric cell testing. The CNO electroplating process is scalable and cost‐effective, making it suitable for industrial applications. This work aims to bridge the gap between high‐performance, lab‐scale ZIBs and cost‐effective, scalable solutions that are essential for commercialization.

## 2 Results and Discussion

**Figure 1:**
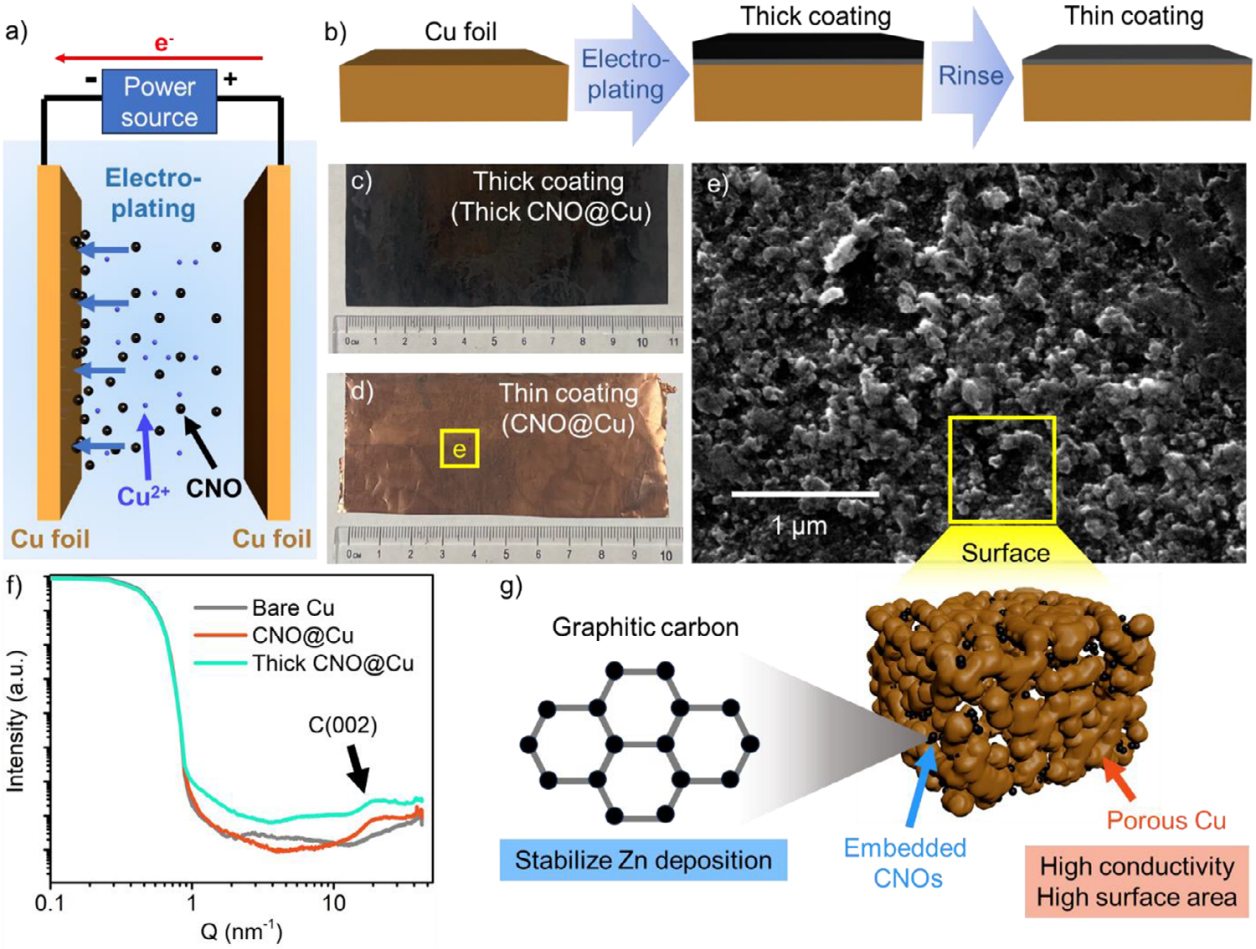
a) Illustrations of the electroplating process. Cu^2+^ and CNOs deposition on Cu foil. b) Schematic of processing steps. Photos of c) thick CNO@Cu and d) CNO@Cu. e) SEM of CNO@Cu. f) GIWAXS profiles of bare Cu, CNO@Cu, and thick CNO@Cu. g) Illustration of CNO@Cu surface, showing the porous Cu structure embedded with CNOs.

We prepared our CNO@Cu by the electroplating method shown in **Figure** **1**a,b. The SEM, HRTEM, and Raman of CNOs are shown in Figures S1 and S2 (Supporting Information). During the electroplating process, both CNOs and Cu were plated on the Cu substrate, resulting in a thick CNO coating on the Cu substrate (Figure 1c; Figure S3, Supporting Information for SEM of cross‐section). Then, the coating was rinsed with DI water to remove the thick outer CNO layer, revealing a thin transparent CNO layer co‐deposited with Cu onto the Cu substrate (Figure 1d). SEM of the surface of the thin coating in Figure 1e revealed CNO particles in a new porous Cu layer on the Cu substrate surface, and the SEM of the cross‐section in Figure S4 (Supporting Information) confirmed that the top CNO layer was washed out. The successful formation of the CNO‐embedded layer was confirmed by the appearance of a distinct C(002) peak at ≈19 nm^−1^ in the GIWAXS patterns (Figure 1f), which is consistent with values reported in the literature.^[^ ^41^, ^42^ ^]^ This peak was absent in the bare Cu sample but observed in both the CNO@Cu and thick CNO@Cu samples, indicating that the thin coating contains embedded CNOs. Furthermore, elemental analysis performed using EDS on the CNO@Cu surface (Table S1, Supporting Information) showed that the presence of carbon was 9.85 wt.%, higher than bare Cu (5.92 wt.%). Although eliminating carbon content during EDS analysis is challenging, as noted in previous studies,^[^ ^43^ ^]^ the comparison between bare Cu and CNO@Cu is a reliable method to confirm the successful deposition of CNO on the Cu surface. The absence of observable carbon XRD peaks for CNO@Cu (Figure S5, Supporting Information) suggested that the CNO‐embedded layer was thin and contained a low amount of CNOs. This is a desirable feature, as it indicates that the coating did not introduce excessive resistance to the current flow, a common issue with coatings with relatively low materials (The conductivity of Cu and CNOs are >10^3^ S cm^−1^ and ≈1 S cm^−1^, respectively^[^ ^35^, ^44^ ^]^). However, increasing the CNO fraction within the porous Cu framework remains a challenge. Future work will focus on enhancing CNO incorporation while preserving the conductive Cu scaffold by optimizing the electrolyte composition (CuCl_2_ and CNO concentrations) and adjusting plating parameters such as voltage and deposition time. Note that the CNO is barely plated on Cu substrate without CuCl_2_ in the electrolyte (Figure S6, Supporting Information), indicating that Cu ions are critical to form a new Cu layer and bind CNO during the electroplating process. Also, numerous studies have shown that graphitic carbon nanomaterials, such as graphene, CNTs, and CNOs, can stabilize zinc deposition.^[^ ^27^, ^29^, ^39^, ^45^ ^]^ Maintaining a thin coating is crucial to preserving the high electrical conductivity of Cu while simultaneously providing nucleation sites for zinc deposition (Figure 1g).

**Figure 2:**
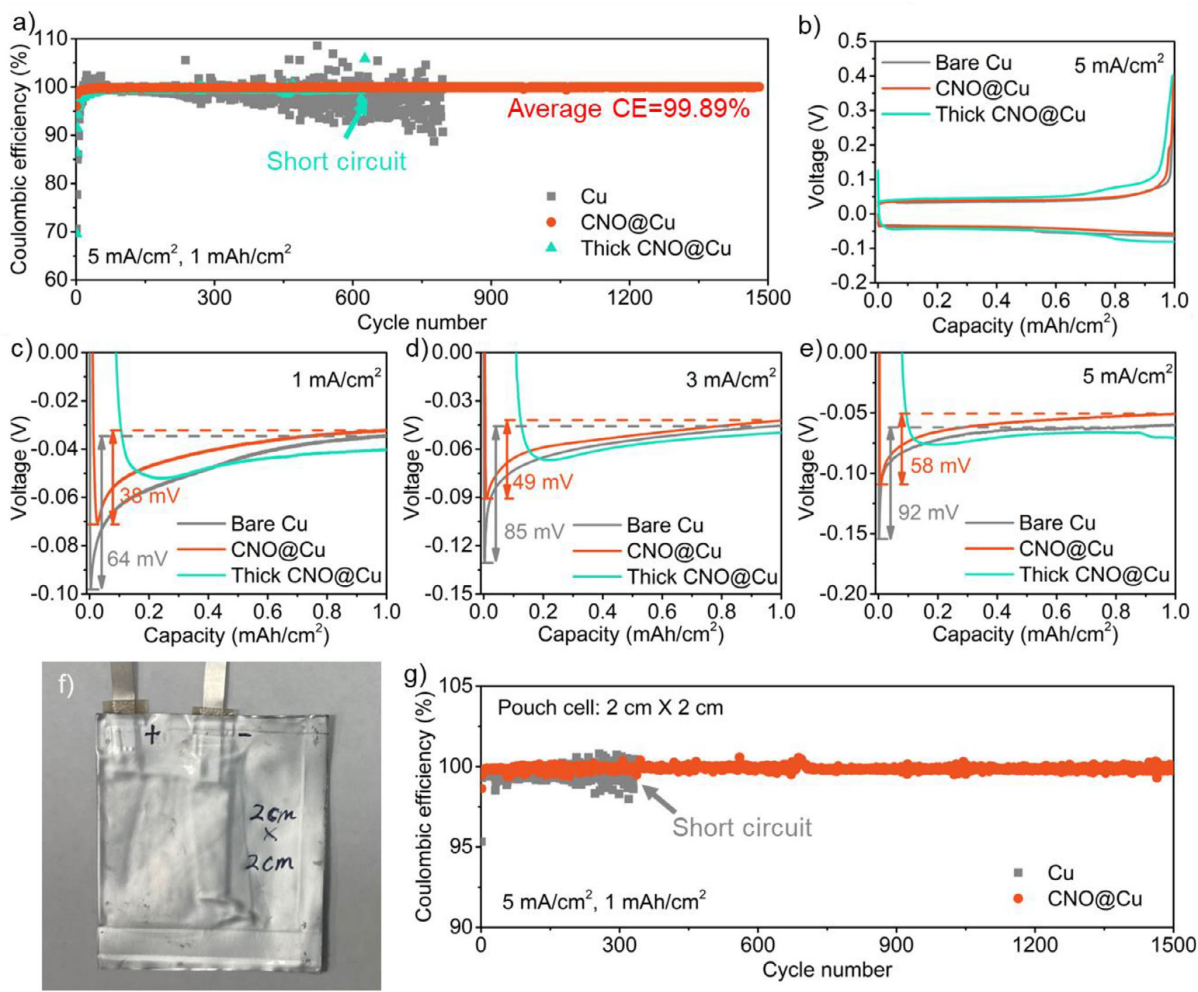
Asymmetric half‐cell test. a) cycling performance for Zn plating/stripping on different electrodes at 5 mA cm^−2^ current density for 1 mAh cm^−2^ capacity. b) voltage profiles for Zn foil || different electrode cells. Nucleation overpotential for Zn plating on different electrodes at c) 1 mA cm^−2^, d) 3 mA cm^−2^, and e) 5 mA cm^−2^. f) Photo of pouch cell with CNO@Cu electrode. Dimension of electrode: 2 cm × 2 cm. g) cycling performance for Zn plating/stripping on different electrodes in pouch cells at 5 mA cm^−2^ current density for 1 mAh cm^−2^ capacity.

We first tested the zinc plating/stripping efficiency by using galvanostatic cycling in an asymmetric cell configuration with CNO@Cu or bare Cu substrate. The results shown in **Figure** **2**a highlight the superior performance of the CNO@Cu. The CE for the first cycle of CNO@Cu reached 96%, significantly higher than the ≈70% CE observed for bare Cu. This initial improvement can be attributed to the uniform zinc nucleation facilitated by the new porous Cu layer, similar to the initial cycle Zn plating/stripping CE (≈96%) for electroplated samples without CNO (Figure S7, Supporting Information), which has a similar porous Cu surface (Figure S8, Supporting Information). In contrast, the Cu plating without CNO resulted in unstable long‐term cycling, underscoring the necessity of the CNO for stabilizing the Zn plating/stripping process. Over subsequent cycles, the CNO@Cu demonstrated remarkable stability, maintaining a high CE of 99.89% throughout the cycling period, while bare Cu exhibited significant performance degradation, with CE dropping rapidly after 200 cycles (Figure 2a). This reduction in CE for bare Cu is due to the uneven zinc deposition, discussed in further detail below. Additionally, Figure S9 (Supporting Information) shows asymmetric cell tests at a current density of 20 mA cm^−2^ with an increased areal capacity of 5 mAh cm^−2^. These results suggest that the CNO@Cu current collector maintains promising cycling performance under higher areal capacity conditions. These results confirm that the CNO@Cu mitigates the issues associated with dendrite formation and passivation, ensuring higher efficiency and longer cycle life.

The effectiveness of the CNO coating was further evaluated by examining the influence of the thick CNO layer on the electrochemical performance of the ZIB. As shown in Figure 2a, the CE of the initial cycle for the thick CNO@Cu was ≈70%, significantly lower than that observed for the CNO@Cu. In overextended cycling, the thick CNO@Cu exhibited a CE of 99.24%, which was inferior to the CNO@Cu and led to a short circuit after ≈620 cycles. Additionally, two coating methods were explored: 1) dispersing CNO in N‐methyl‐2‐pyrrolidone (NMP) followed by drop casting onto Cu and drying at 80 °C for 12 h, and 2) blending CNO and polyvinylidene fluoride (PVDF) in a 9:1 ratio in NMP, drop casting onto Cu, and drying under the same conditions. Both methods demonstrated suboptimal performance, with initial CEs of 91.92% and 93.33%, respectively, and unstable long‐term cycling (Figure S10, Supporting Information), indicating that conventional coating techniques with CNOs do not provide an ideal substrate for ZIB anodes. The voltage profile (Figure 2b) revealed that thick CNO@Cu displayed higher stripping voltages than both CNO@Cu and bare Cu, particularly at capacities approaching 1 mAh cm^−^^2^, suggesting that the lower conductivity of the thick CNO layer impedes electron flow uniformity.

Figure 2c–e presents the nucleation overpotentials at different current densities (1, 3, and 5 mA cm^−2^) for bare Cu, CNO@Cu, and thick CNO@Cu. For the CNO@Cu, the nucleation overpotential at 1 mA cm^−^^2^ was measured to be 38 mV, significantly lower than the 64 mV observed for bare Cu. This reduction in nucleation overpotential indicates that the CNO@Cu surface provides more favorable conditions for uniform zinc nucleation, reducing the energy barrier for zinc ion deposition. Interestingly, the thick CNO coating displayed the lowest nucleation overpotential, suggesting that thicker carbon layers can further moderate zinc nucleation. However, the increased resistance observed during the later stages of plating (made evident by more negative voltages in the steady‐state plating phase) indicated that the thick coating impeded the uniform flow of electrons, leading to increased plating energy.

Subsequently, the Zn plating/stripping performance was investigated on larger substrates (2 cm × 2 cm) with electrochemical testing in a pouch cell configuration (Figure 2f). The initial cycle CE for the CNO@Cu was 98.62%, surpassing the 95.32% achieved with bare Cu (Figure 2g), indicating the scalability and potential applicability of the CNO@Cu for larger‐scale ZIBs. Note that improved first‐cycle CE for pouch cell is likely due to the higher actual current, rather than differences in sealing or configuration (Figure S11, Supporting Information). Long‐term cycling performance in the pouch cell demonstrated that the CNO@Cu effectively mitigates issues related to dendritic growth and electrode passivation, highlighting the promise of CNO@Cu as a robust anode current collector for high‐performance, large‐format ZIBs.

**Figure 3:**
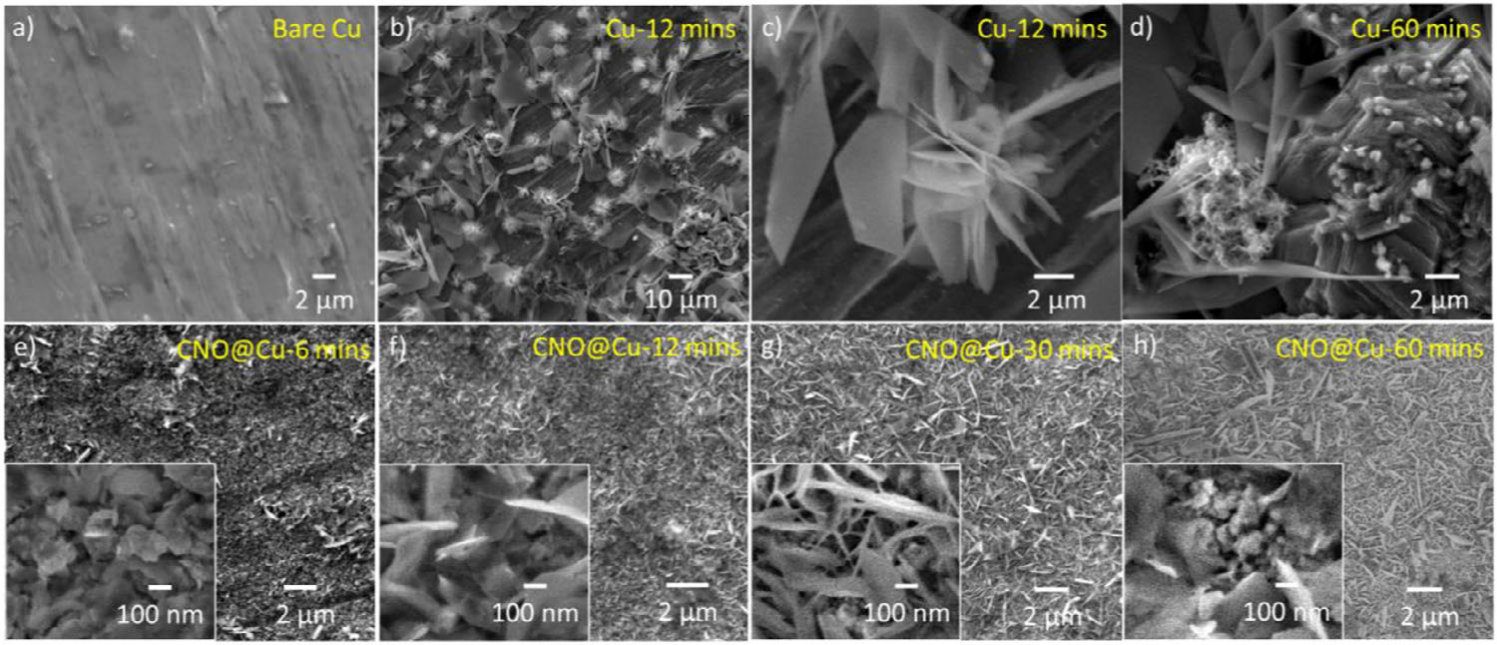
Zinc plating on Cu or CNO@Cu at current density: 5 mA cm^−2^. SEM of a) Bare Cu; b) and c) Cu for 12 min; d) Cu for 60 min; e) CNO@Cu for 6 min; f) CNO@Cu for 12 min; g) CNO@Cu for 30 min; and h) CNO@Cu for 60 min.

To understand the impact of CNO@Cu on zinc morphology, we conducted detailed SEM analysis of zinc‐plated CNO@Cu and Cu samples after different durations of plating. SEM images (**Figure** **3**a–d) clearly show that, after only 12 min of zinc plating on bare Cu, flower‐like zinc began to form, with plate sizes exceeding 20 microns. These large, irregular structures indicate a highly uneven zinc deposition process, which is a hallmark of dendrite growth. Such dendritic structures, if left unchecked, can penetrate the separator over time, causing short circuits and a short lifespan of the battery. In contrast, the zinc plating on CNO@Cu was significantly more uniform, as seen in the smaller and more evenly distributed zinc deposits (Figure 3e–h). The graphitic structure of the CNOs promotes zinc ions to uniformly nucleate and grow, as previously reported in the literature.^[^ ^27^, ^39^ ^]^ After 60 min, the Zn deposits on CNO@Cu remain compact and uniform, without large Zn plates or dendritic structures, and XRD analysis in Figure S12 (Supporting Information) displays only peaks corresponding to Zn and Cu, with no detectable signals from ZnO,^[^ ^46^ ^]^ ZnOH,^[^ ^47^ ^]^ or other byproducts, indicating that the CNO‐embedded layer effectively suppresses dendrite formation even under extended plating conditions. Also, Figure S13 (Supporting Information) shows that some residual Zn can be observed on the CNO@Cu surface after 100 Zn plating/stripping cycles, but there is no sign of structural collapse.

**Figure 4:**
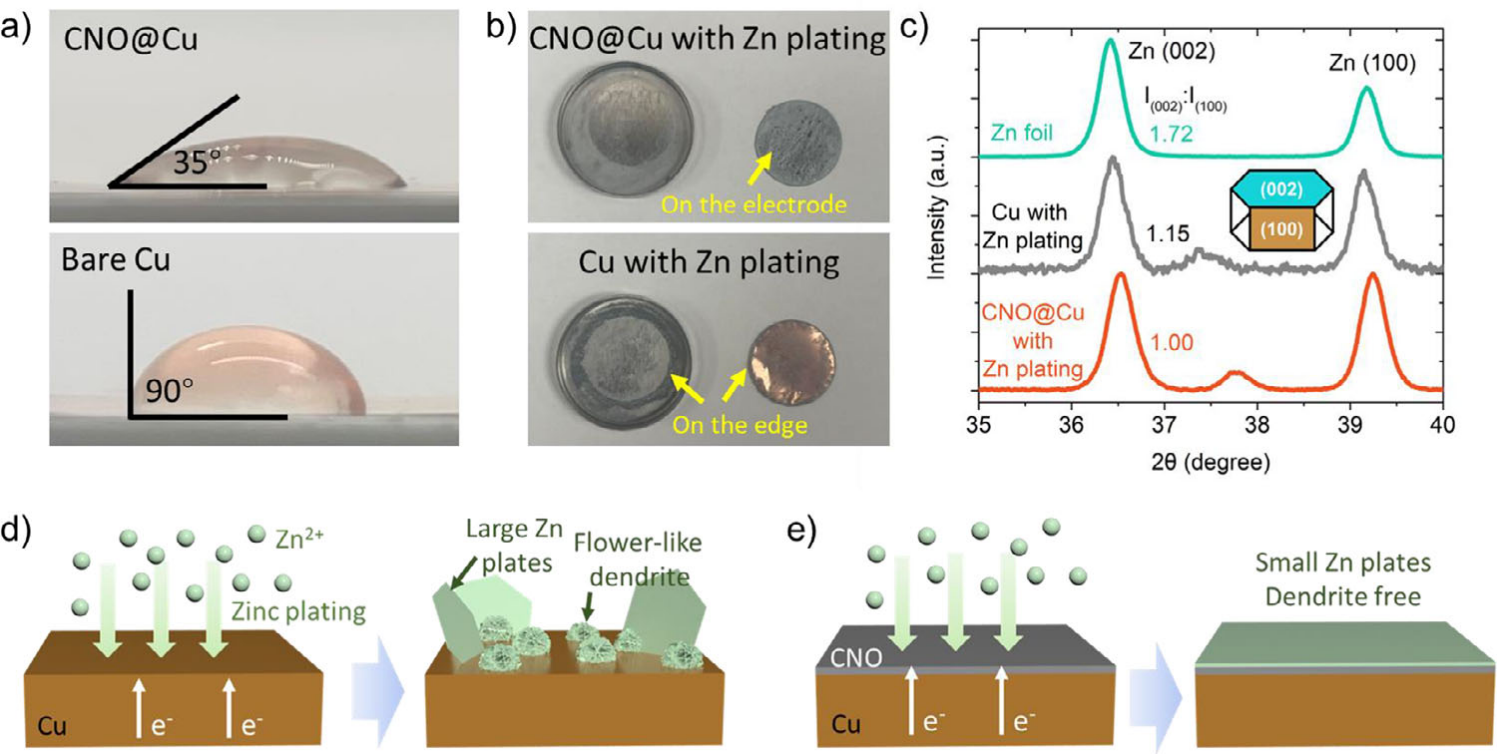
a) Contact angle measurement for CNO@Cu and Cu foil; b) Photo of coin cell case and CNO@Cu or Cu electrodes with 1 mAh cm^−2^ Zn plating at 5 mA cm^−2^; c) XRD for Zn foil, Cu with Zn plating, and CNO@Cu with Zn plating (1 mAh cm^−2^ at 5 mA cm^−2^); Schematic for zinc plating on d) Cu and e) CNO@Cu.

To better understand the underlying mechanism, we measured the contact angles of water with bare Cu and CNO@Cu (**Figure** **4**a), which were 90° and 35°, respectively. The lower contact angle of CNO@Cu is attributed to the rougher surface of the CNO‐embedded layer (Figure 1e), enhancing its hydrophilicity. Linear sweep voltammetry for CNO@Cu in Figure S14 (Supporting Information) indicates that the CNO@Cu surface shows negligible hydrogen evolution activity under battery‐relevant conditions. The Tafel plot (Figure S15, Supporting Information) shows that the corrosion current density of the CNO@Cu electrode is on the order of 10^−8^ A cm^−2^, suggesting a low corrosion rate in ZnSO_4_ electrolyte. Figure 4b shows that the increased hydrophilicity promotes preferential Zn deposition on the CNO@Cu surface, whereas Zn primarily deposits along the edges of bare Cu (which can be considered inactive or “dead” zinc), as the aqueous ZnSO_4_ electrolyte favors hydrophilic surfaces. By reducing the number of inactive or “dead” zinc sites formed during deposition, the CNO@Cu minimizes irreversible zinc losses and enhances the reversibility of the plating/stripping process. To further investigate the Zn deposition behavior, we conducted XRD analysis on Zn‐plated Cu and CNO@Cu electrodes (Figure 4c). We focus on the 35°–40° range, with the full XRD pattern provided in Figure S16 (Supporting Information). The characteristic Zn peaks include Zn(002) at 36.3°, Zn(100) at 39.1°, and Zn(101) at 43.3°.^[^ ^48^ ^]^ However, Zn(101) overlaps with Cu(111), complicating its direct observation.^[^ ^49^ ^]^ More critically, the intensity ratio I_Zn(002)_/I_Zn(100)_ is 1.00 for Zn‐plated CNO@Cu, indicating polycrystalline Zn deposition without a preferred orientation. In contrast, the higher I_Zn(002)_/I_Zn(100)_ ratio for Zn‐plated Cu suggests a preferential Zn(002) orientation, in agreement with SEM observations in Figure 3. Larger Zn platelets with a high aspect ratio (002‐oriented over 100‐oriented Zn) are observed on Cu, leading to dendritic growth. Conversely, CNO@Cu promotes smaller Zn plates with lower aspect ratios, resulting in a more uniform Zn coating. The observed uniform Zn morphology on CNO@Cu (Figure 3e–h) indicates that Zn nucleation occurs across the composite surface rather than concentrating at isolated regions. The low contact angle (35°, Figure 4a) confirms enhanced electrolyte wettability, suggesting Zn preferentially nucleates on CNO‐rich areas. Given the embedded structure, Zn likely deposits at the CNO‐Cu interface, where graphitic surfaces promote uniform nucleation while maintaining electron conduction via the Cu matrix. This distributed nucleation mechanism contrasts with the edge‐focused or dendritic growth seen on bare Cu (Figure 3a–d).

Figure 4d,e illustrates our proposed explanation for Zn plating on Cu and CNO@Cu, respectively. For Zn plating on Cu current collectors, we have observed large Zn plates, dendrite formation, and non‐uniform Zn deposition, whereas uniform Zn plating on the CNO@Cu substrate shows that CNO@Cu effectively mitigates dendrite formation, enhancing the longevity and reliability of ZIBs. Our porous CNO@Cu surface suppresses dendrites by regulating Zn deposition size and promoting compact deposition, rather than inducing preferential orientation and stacking of large Zn flakes as reported in previous studies.^[^ ^50^, ^51^ ^]^

**Figure 5:**
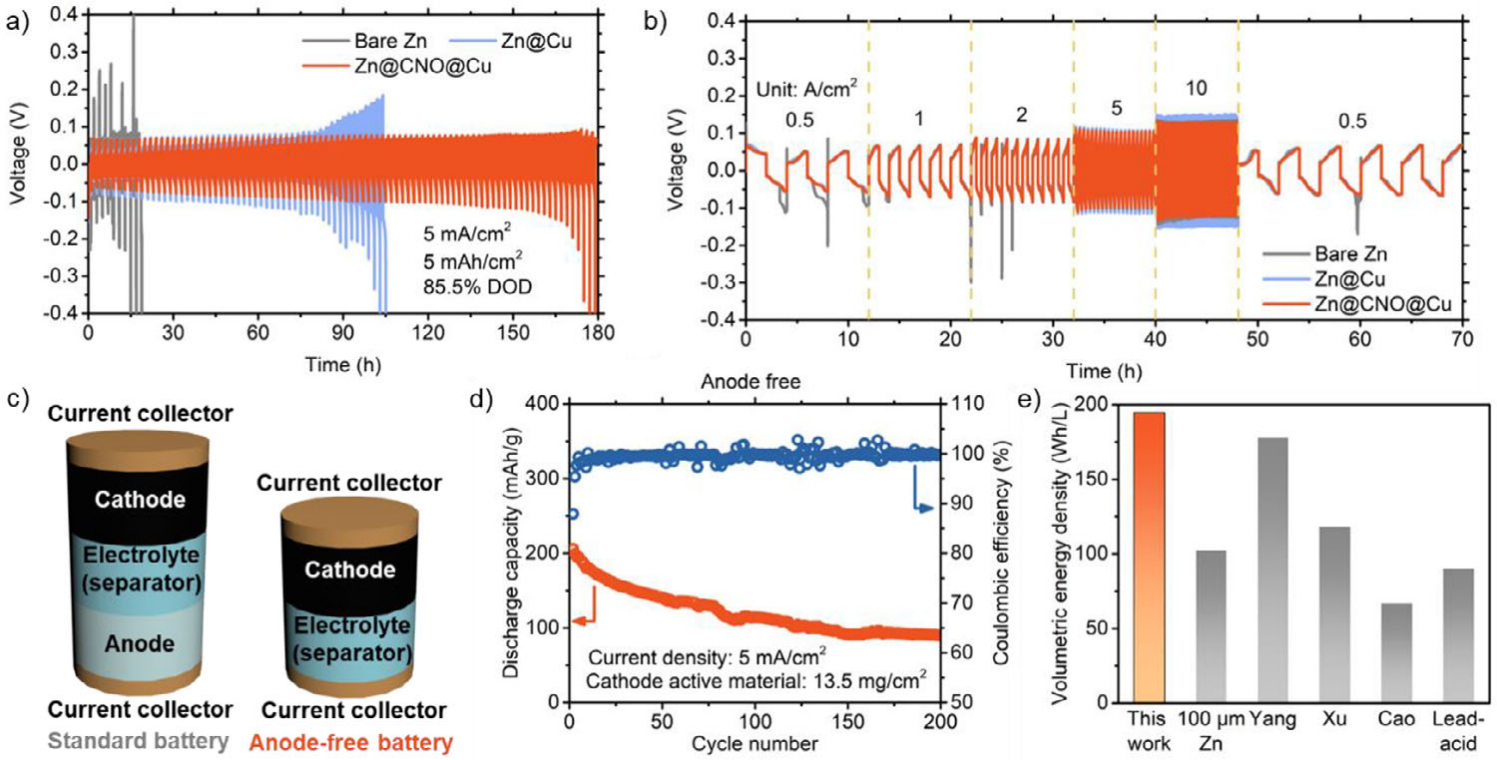
Symmetrical‐cell and full‐cell tests. a) voltage profiles for 10 µm Zn foil, 10 µm Zn@Cu, and 10 µm Zn@CNO@Cu symmetric cells test at 5 mA cm^−2^ with 5 mAh cm^−2^ capacity. The capacity of 10 µm Zn foil is 5.88 mAh cm^−2^, and the DOD is 85.5% in this test. b) voltage profiles for 10 µm Zn foil, 10 µm Zn foil on Cu (Zn@Cu) and 10 µm Zn foil CNO@Cu (Zn@CNO@Cu) symmetric cells test at different current densities with 1 mAh cm^−2^ capacity. c) Schematic of anode‐free batteries. d) Cycling performance for high‐loading (cathode material loading: 13.5 mg cm^−2^) anode‐free battery at 5 mA cm^−2^ current density. e) Volumetric energy densities of Zn‐ion batteries for this work (orange bars) and others (gray bars; our 100 µm Zn foil control, Lead acid,^[^ ^58^ ^]^ Yang et al.,^[^ ^28^ ^]^ Xu et al.,^[^ ^59^ ^]^ Cao et al.^[^ ^60^ ^]^).

The long‐term cycling stability of the CNO@Cu as a current collector was evaluated in symmetric cells using 10 µm zinc foil as the electrode. **Figure** **5**a compares the cycling performance of bare Zn, Zn on Cu, and Zn on CNO@Cu at a current density of 5 mA cm^−2^ with an 85.5% DOD. The bare Zn cell failed after just 10 h of cycling, which may be due to severe dendrite formation, whereas the Zn on Cu cell exhibited a cycle life of 102 h, consistent with previous literature.^[^ ^28^ ^]^ Remarkably, the Zn on CNO@Cu cell demonstrated a cycle life of 176 h, nearly double that of Zn on Cu. This extended cycle life is directly attributable to the CNO‐embedded layer's ability to promote uniform zinc deposition and prevent dendrite formation. The lower voltage polarization observed for the CNO@Cu (Figure 5b) further suggests that the coating improves the rate capability of the battery. Figure S17 (Supporting Information) presents the symmetric cell tests with 51.0% DOD for bare Zn, Zn@Cu, and Zn@CNO@Cu electrodes (25 µm zinc foil was used in this test), conducted at a current density of 5 mA cm^−2^ and a high capacity of 7.5 mAh cm^−2^. The bare Zn electrode lasted 69 h, while Zn@Cu failed after 87 h. Zn@CNO@Cu survived 165 h, which is almost double the lifespan of Zn@Cu, demonstrating that CNO@Cu is significantly more effective as a current collector for ZIB anodes than bare Cu. Also, Figure S18 (Supporting Information) shows symmetric cell tests at a lower current density of 1 mA cm^−2^, using 1 mAh cm^−2^ areal capacity, and the Zn@CNO@Cu symmetric cells demonstrated extended cycle life (more than 400 h). Figure S19 (Supporting Information) further compares the performance of different current collectors in symmetric cell tests. The results show that electroplating without CNO in the electrolyte (lasting 81 h at 85.5% DOD) and without CuCl_2_ (lasting 107 h at 85.5% DOD) perform similarly to bare Cu but are inferior to our CNO@Cu. This confirms that the CNO@Cu, with its ultra‐thin CNO‐embedded layer, is highly effective for the ZIB anode.

We investigate our CNO@Cu in the full ZIB configurations with NH_4_V_4_O_10_ cathode. Figure S20 (Supporting Information) shows the performance of standard ZIBs with Zn foil or zincified CNO@Cu. When CNO@Cu was applied as the anode current collector, the capacity retention increased. For example, the capacity retention of 100th and 500th cycles for Zn@CNO@Cu cell is 96.75% and 46.10%, higher than 82.50% and 31.75% for Zn foil cell. This performance demonstrates that the CNO@Cu can stabilize the anode and improve the capacity retention of the full cell.

We also employed anode‐free configurations (Figure 5c) to test our CNO@Cu as an anode current collector. Anode‐free ZIBs have garnered interest due to their potential to simplify battery construction and increase energy density.^[^ ^45^, ^52^, ^53^, ^54^, ^55^ ^]^ However, their success relies heavily on the efficiency of the zinc plating/stripping process, as there is no excess zinc available to compensate for inefficiencies. Note that we discharged our cathode material to zincify it before using it in the anode‐free cell (XRD of zincified NH4V4O10 in Figure S21, Supporting Information). The anode‐free cell exhibited comparable performance to the standard cell in the initial cycles (comparing Figure S22 with Figure S20, Supporting Information), with similar charge–discharge voltages and capacity retention. We also tested with high cathode material loading (13.5 mg cm^−^^2^) in Figure 5d, the anode‐free cell exhibited a specific capacity of 204 mAh g^−1^ in the 2nd cycle, similar to that of the low‐loading cell in Figure S20 (Supporting Information). After 100 and 200 cycles, the cell maintained 56% and 45% of its initial capacity, respectively, demonstrating acceptable stability given the high material loading. Nonetheless, the CNO@Cu anode‐free cell achieved a volumetric energy density of 194 Wh L^−1^ (Table S2, Supporting Information), significantly higher than the 90 Wh L^−1^ typical of lead‐acid batteries (Figure 5e). The gravimetric energy density of our anode‐free cell is 84 Wh kg^−1^, and for comparison, recently reported values for anode‐free Zn batteries are 86 and 135 Wh kg^−1^.^[^ ^45^, ^56^ ^]^

## 3 Conclusion

The ability to achieve such high volumetric energy density using a low‐cost, industrially‐scalable, CNO@Cu current collector suggests that this technology has the potential to meet the demands of high‐energy‐density applications with an economically viable nanomaterial. In conclusion, the CNO@Cu as ZIB anode current collectors developed in this study offer a scalable and cost‐effective solution for stabilizing the zinc anode. The ultra‐thin, transparent CNO‐embedded‐Cu layer on Cu enhances zinc plating/stripping efficiency (99.89% at 5 mA cm^−2^ with 1 mAh cm^−2^), suppresses dendrite formation, and improves long‐term cycling stability, making it a promising candidate for commercial ZIB applications. Additionally, the CNO‐embedded‐Cu layer ensures high conductivity and promotes zinc depositions with graphitic carbon nanomaterials. The CNO@Cu exhibited superior cycling performance, maintaining an 85.5% depth of discharge over 176 h, compared to bare Cu, which lasted only 102 h under the same conditions. Furthermore, in an anode‐free configuration paired with an NH_4_V_4_O_10_ cathode, the battery demonstrated a high volumetric energy density of 194 Wh L^−1^. Our CNO@Cu provides a cost‐effective, scalable, and high‐performance solution for industrial ZIB applications, and this study lays the groundwork for future advancements in ZIBs through the use of affordable, industrial‐grade nanomaterials.

## 4 Experimental Section

### Materials

CNO (synthesized by Nabors Industries), Cu foil (thickness: 9 µm, >99.8%, Beyond Battery), Zn foil (thickness: 10 µm, >99.9%, purchased from Amazon; thickness: 25 µm, >99.9%, MSE supplies; thickness: 200 µm, >99.9%, purchased from Mc‐McMaster‐Carr), CuCl_2_ (anhydrous, reagent grade, Innovative Science), Hexadecyltrimethylammonium bromide (CTAB, ≥98%, Sigma‐Aldrich), 2‐Amino‐2‐(hydroxymethyl)‐1,3‐propanediol (Trisma base, ≥99.7%, Sigma–Aldrich), HCl (37%, Sigma–Aldrich), 2‐Propanol (IPA, >99.9%, Honeywell), deionized water (DI water, > 18 MΩ cm^−1^), glass fiber filter (Grade: GA55, porous size: 0.6 µm, Advantec MFS), ZnSO_4_·7H_2_O (ACS reagent, 99%, Sigma–Aldrich), NH_4_VO_3_(ACS reagent, ≥99.0%, Sigma–Aldrich), H_2_C_2_O_4_·2H_2_O (ACS reagent, ≥99%, Sigma–Aldrich), super C65 carbon black (Super P, particle size< 50 nm, MSE Supplies), poly(vinylidene fluoride) (PVDF, average Mw ≈534 000, Sigma–Aldrich), 1‐Methyl‐2‐pyrrolidinone (NMP, anhydrous, 99.5%, Sigma–Aldrich), carbon paper (PTFE treated, TGP‐H‐60, Thermo Scientific). The CNO was exposed to ozone to increase hydrophilicity prior to electroplating.

### Electroplating Method

The electrolyte was prepared by mixing 400 mL of DI water, 4 g of CNOs, 150 mg of CuCl_2_, 300 mg of CTAB, 4 mL of 1 m Tris buffer solution, and 3 mL of 1 m HCl solution (adjusted to pH 5.0). This mixture was stirred for 30 min and then bath sonicated @37 kHz for an additional 30 min. Two Cu foils (dimensions: 150 mm × 190 mm) were fully immersed in the electrolyte and connected to the positive and negative poles of the power supply. A voltage of 3 V was applied for 1 h, during which the initial current of ≈650 mA gradually dropped to 300 mA. After the electroplating process, the Cu foil serving as the negative electrode was thoroughly rinsed with DI water to obtain the CNO@Cu sample. If the final DI water rinsing step was omitted, a thicker CNO@Cu sample was obtained.

### NH_4_V_4_O_10_ Cathode Preparation

The synthesis of NH_4_V_4_O_10_ followed a previously reported method.^[^ ^57^ ^]^ First, 1.170 g of NH_4_VO_3_ was added to 35 mL of DI water and stirred at 80 °C until fully dissolved. Then, 1.891 g of H_2_C_2_O_4_·2H_2_O was slowly added while stirring, resulting in a dark blue‐green solution. This solution was transferred into a 50 mL autoclave and heated in an oven at 140 °C for 48 h. After cooling, the resulting material was collected, thoroughly washed with DI water, and dried in a vacuum oven at 90 °C for 12 h. Preparation of cathode electrode: The cathode electrode was prepared by mixing NH_4_V_4_O_10_, Super P, and PVDF at a mass ratio of 7:2:1 in NMP solvent. The resulting mixture was uniformly drop‐cast onto carbon paper and dried in a vacuum oven at 80 °C for 12 h. The effective mass loading of the active material was ≈1.5 and 13.5 mg cm^−2^.

### Electrochemical Test

A 100 µL 2 m ZnSO_4_ solution and a glass fiber filter were used as the electrolyte and separator, respectively, to assemble coin cells. A 200 µL 2 m ZnSO_4_ solution and a glass fiber filter were used as the electrolyte and separator, respectively, to assemble pouch cells. All cells were rested for 12 h before testing. For the half‐cell tests, a circular or square Zn foil (12.7 mm diameter for coin cell; 2 cm × 2 cm for pouch cell) with 200 µm thickness was used as the anode, while various circular or square substrates (9.5 mm diameter for coin cell; 2 cm × 2 cm for pouch cell) were used as the cathode to assemble coin cells (type 2032) and pouch cells. For the symmetric‐cell tests, 10 µm Zn foils on different substrates were used as electrodes (diameter: 12.7 mm). A cold press (≈50 kPa) was applied to attach the Zn foil to the various substrates. For full‐cell tests, either a 200 µm Zn foil or pre‐zincified CNO@Cu was used as the anode (diameter: 12.7 mm) to assemble coin cells (type 2025) with NH_4_V_4_O_10_ as the cathode (diameter: 9.5 mm). For the anode‐free battery, the NH_4_V_4_O_10_ cathode (diameter: 9.5 mm) was pre‐discharged in a coin cell with a Zn foil anode. The coin cell was then disassembled to obtain the zincified NH_4_V_4_O_10_ cathode. Before assembling the anode‐free battery, the zincified NH_4_V_4_O_10_ electrode was thoroughly washed with DI water and dried at 80 °C overnight. The anode‐free battery was assembled using CNO@Cu as the anode (diameter: 12.7 mm) and zincified NH_4_V_4_O_10_ as the cathode in coin cells (type 2025). Galvanostatic charge–discharge tests were performed using a LANHE CT3002A system.

### Material Characterization

SEM: JEOL JSM‐7500F and FEI QUANTA 600 were used to observe the morphologies of the samples. Each sample was sputter coated with a 5‐nanometer thick layer of Pt/Pd using a sputter coater (Cressington 208HR) before SEM measurement. XRD was characterized by a Bruker D2 Phaser within a scanning range of 5°–120°, with a step size of 0.02° and a rate of 1.2° min^−1^. GIWAXS was performed using a small‐angle X‐ray scattering (Xenocs Xeuss 3.0 system, France) with the GeniX 3D X‐ray beam delivery system, employing a sample‐to‐detector distance of 75 mm and an exposure time of 240 s.

## Conflict of Interest

Nabors authors acknowledge intellectual property holdings on the CNO synthesis and electroplating process referenced here. Nabors funded much of the work carried out on this topic at TAMU.

## Supporting information



Supporting Information

## Data Availability

The data that support the findings of this study are available from the corresponding author upon reasonable request.
