# Supplementary material for: Electroplating Carbon Nano‐Onion on Copper for Dendrite‐Free and Anode‐Free Zinc‐Ion Batteries
Source: Adv Sci (Weinh). 2025 Aug 13;12(42):e10617. doi: 10.1002/advs.202510617 (PMC12622529; doi:10.1002/advs.202510617)
Supplement: Supplementary file 1 — Supporting Information [file ADVS-12-e10617-s001.docx]

**SUPPLEMENTARY INFORMATION**

**Electroplating Carbon Nano-Onion on Copper for Dendrite-Free and Anode-Free Zinc-Ion Batteries**

Yufan Zhang^1^, Ramu Banavath^1^, Shegufta Upama^2^, Sayyam Deshpande^1^, Huaixuan Cao^1^, Brian R. Stepp^3^, Navid Attarzadeh^3^, Stephnie Peat^3^, Joseph V. Kosmoski^3^, Evan C. Johnson^3^, Micah J. Green^1,2 *^

1 Artie McFerrin Department of Chemical Engineering, Texas A&M University, College Station, Texas, 77843 USA

2 Department of Material Science and Engineering, Texas A&M University, College Station, Texas, 77843 USA

3 Nabors Energy Transition Solutions LLC, Houston, Texas, 77067 USA

*Corresponding author: micah.green@tamu.edu


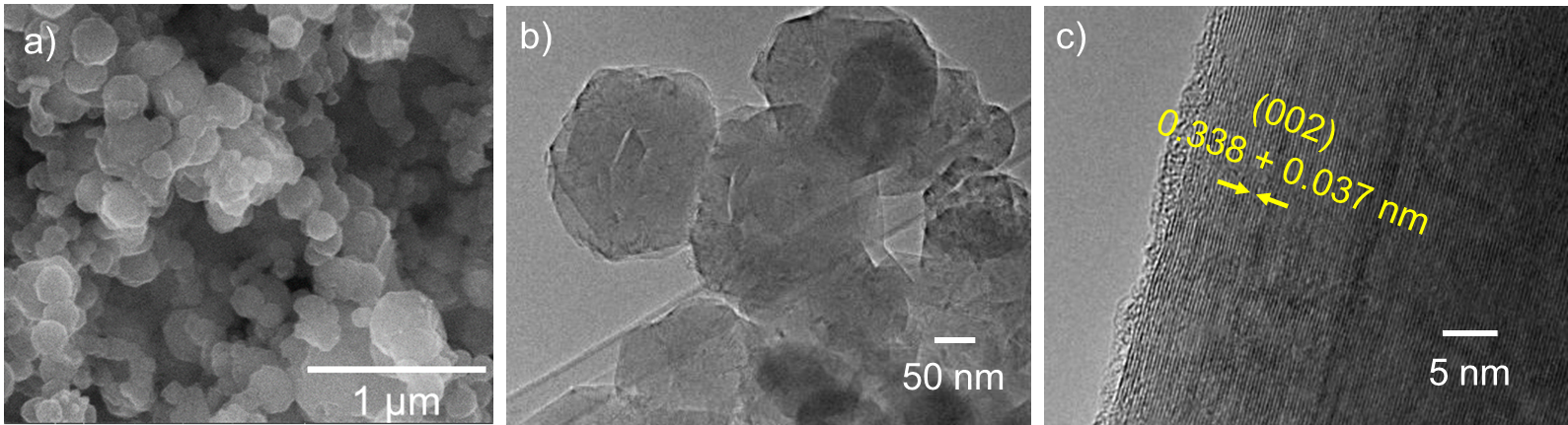


Figure S1. SEM of carbon nano-onions (CNO). The material shows a pre-networked structure. High-resolution transmission electron micrographs of (b-c) CNO. Reproduced from Zhang *et al.*^1^


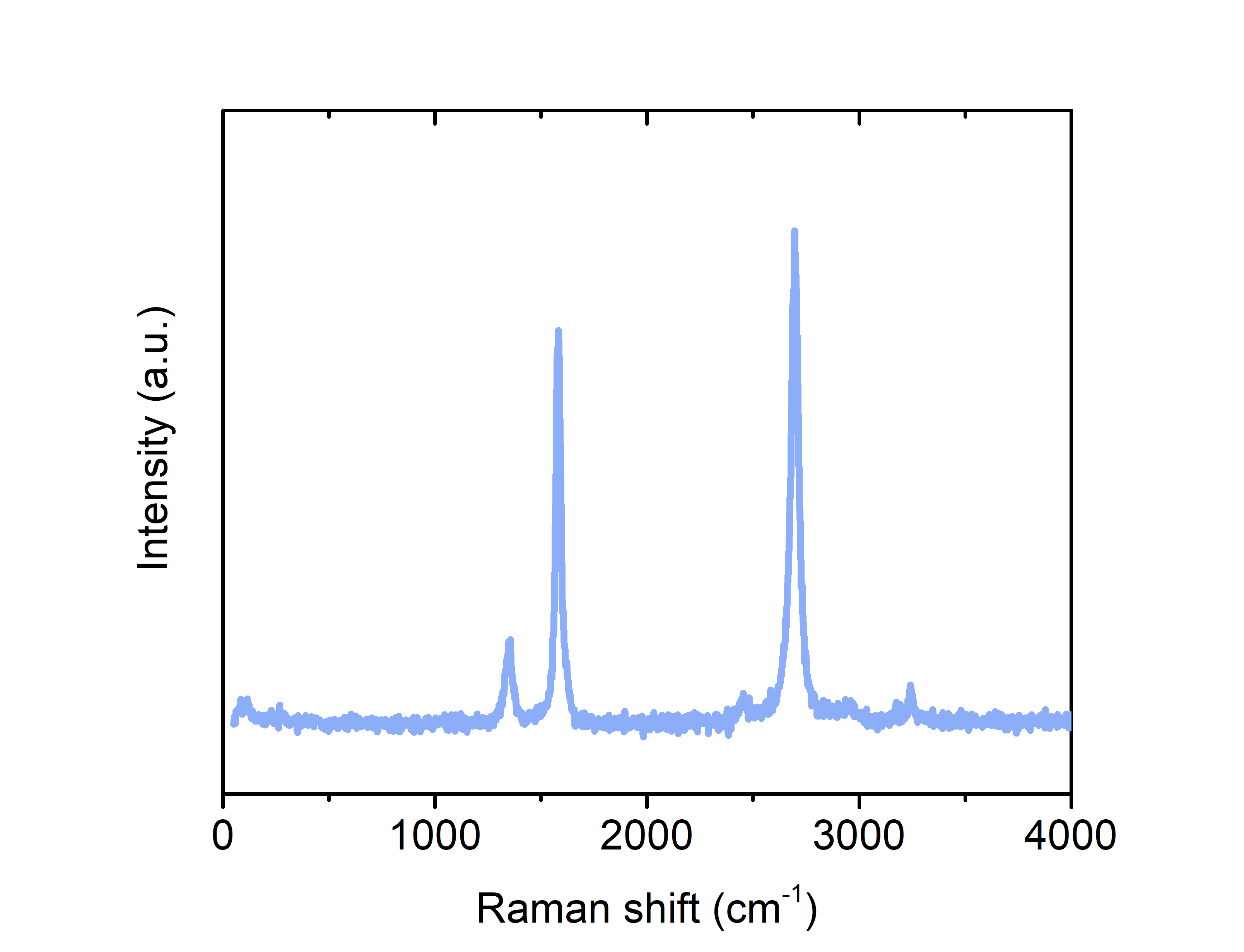


Figure S2. Raman of CNO. D band at 1350 cm^-1^, G band at 1570 cm^-1^, and 2D band at 2700 cm^-1^. The I_D_/I_G_ ratio based on the intensities for CNO is 0.22. Reproduced from Zhang *et al*.^1^


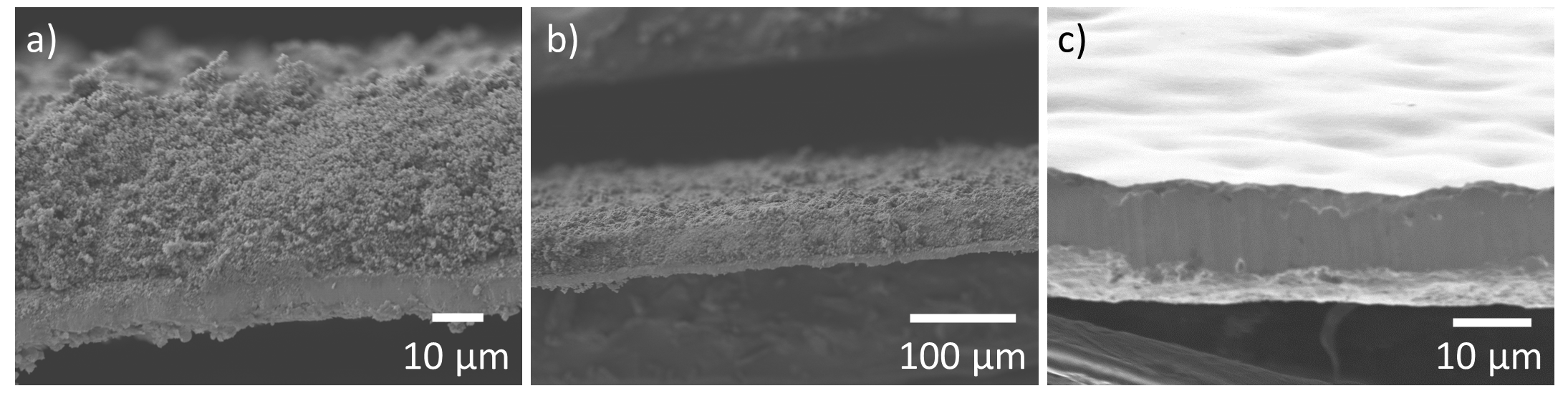


Figure S3. SEM of cross section of (a-b) thick CNO@Cu, and (c) bare Cu. The thickness of Cu foil is ~9 microns. The thickness of CNO coating is ~30-35 microns.


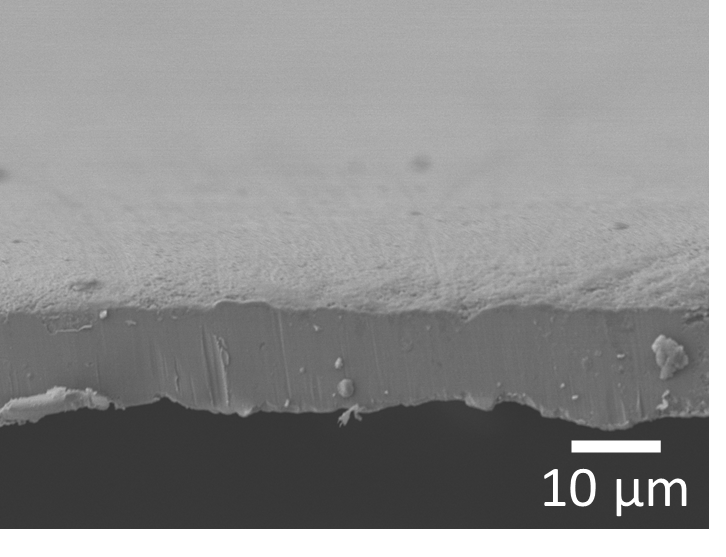


Figure S4. SEM of cross section of CNO@Cu.


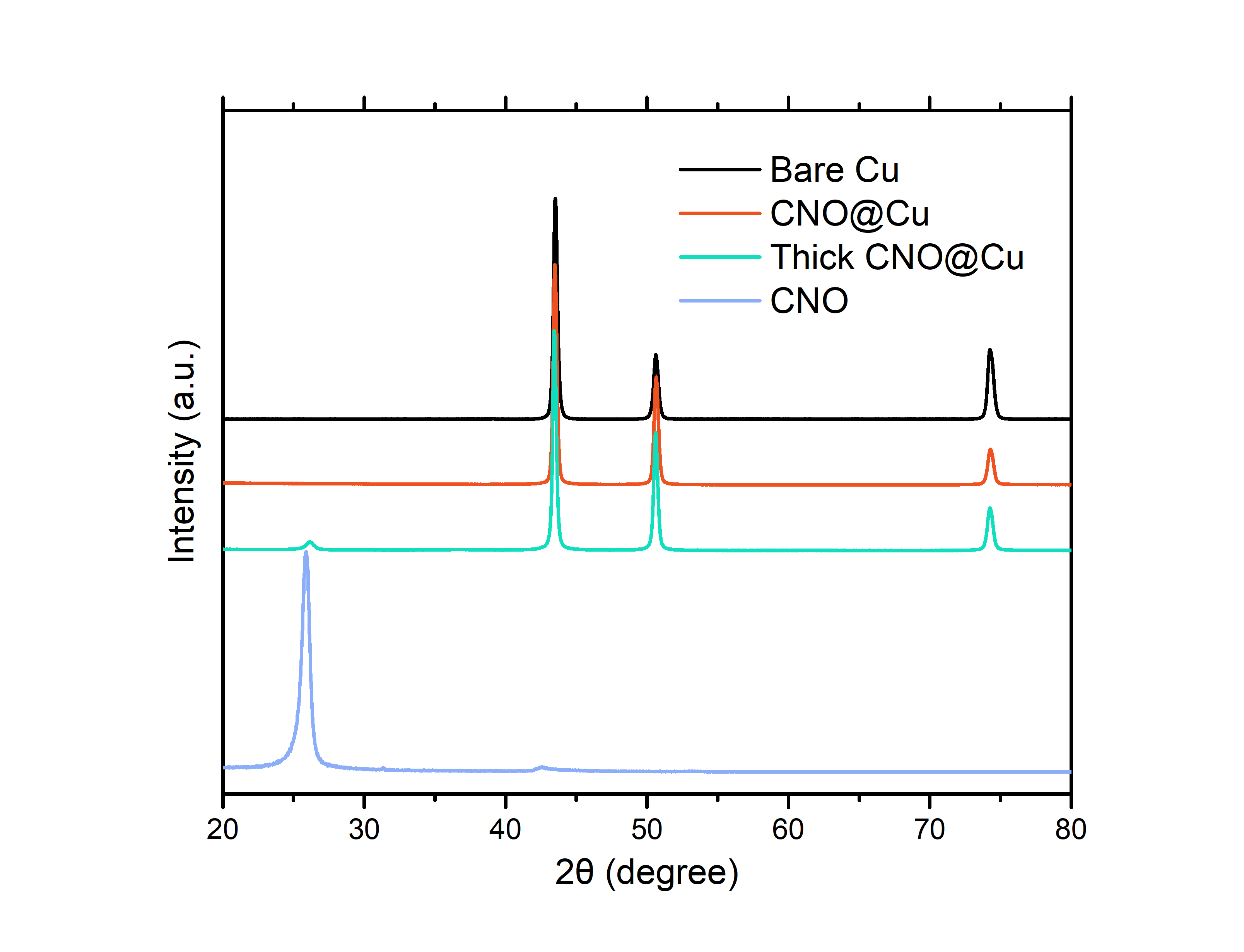


Figure S5. XRD of bare Cu, CNO@Cu, thick CNO@Cu, and pristine CNO

Table S1. EDS of different samples (for top surface)

| Element | Bare Cu | E-plating Cu without CuCl_2_ in the electrolyte | CNO@Cu |
| --- | --- | --- | --- |
| Cu | 94.08 wt% | 93.65 wt% | 90.15 wt% |
| C | 5.92 wt% | 5.46 wt% | 9.85 wt% |
| O | 0 wt% | 0.89 wt% | 0 wt% |


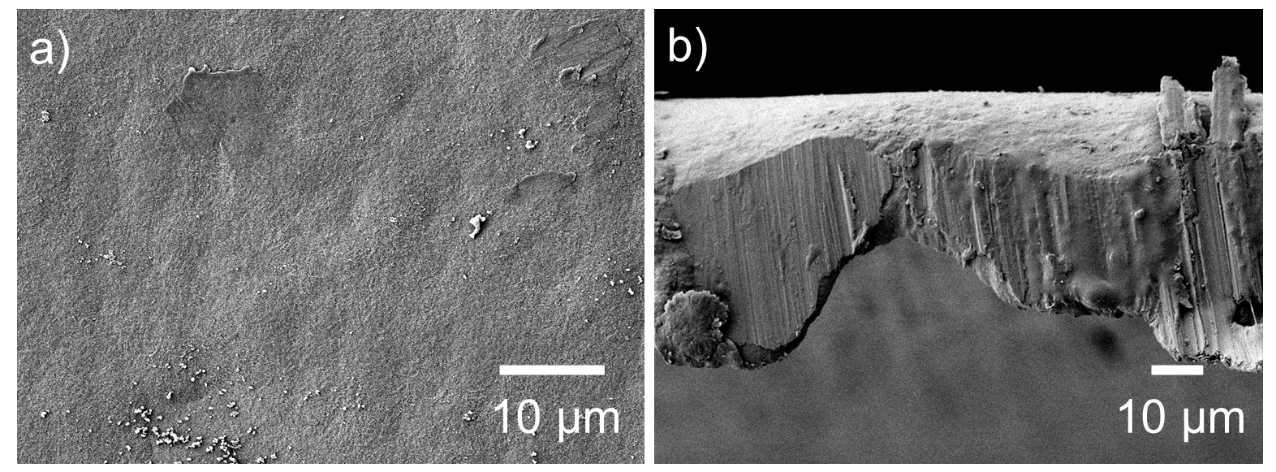


Figure S6. SEM of (a) surface and (b) cross-section of electroplating Cu without CuCl_2_ in the electrolyte


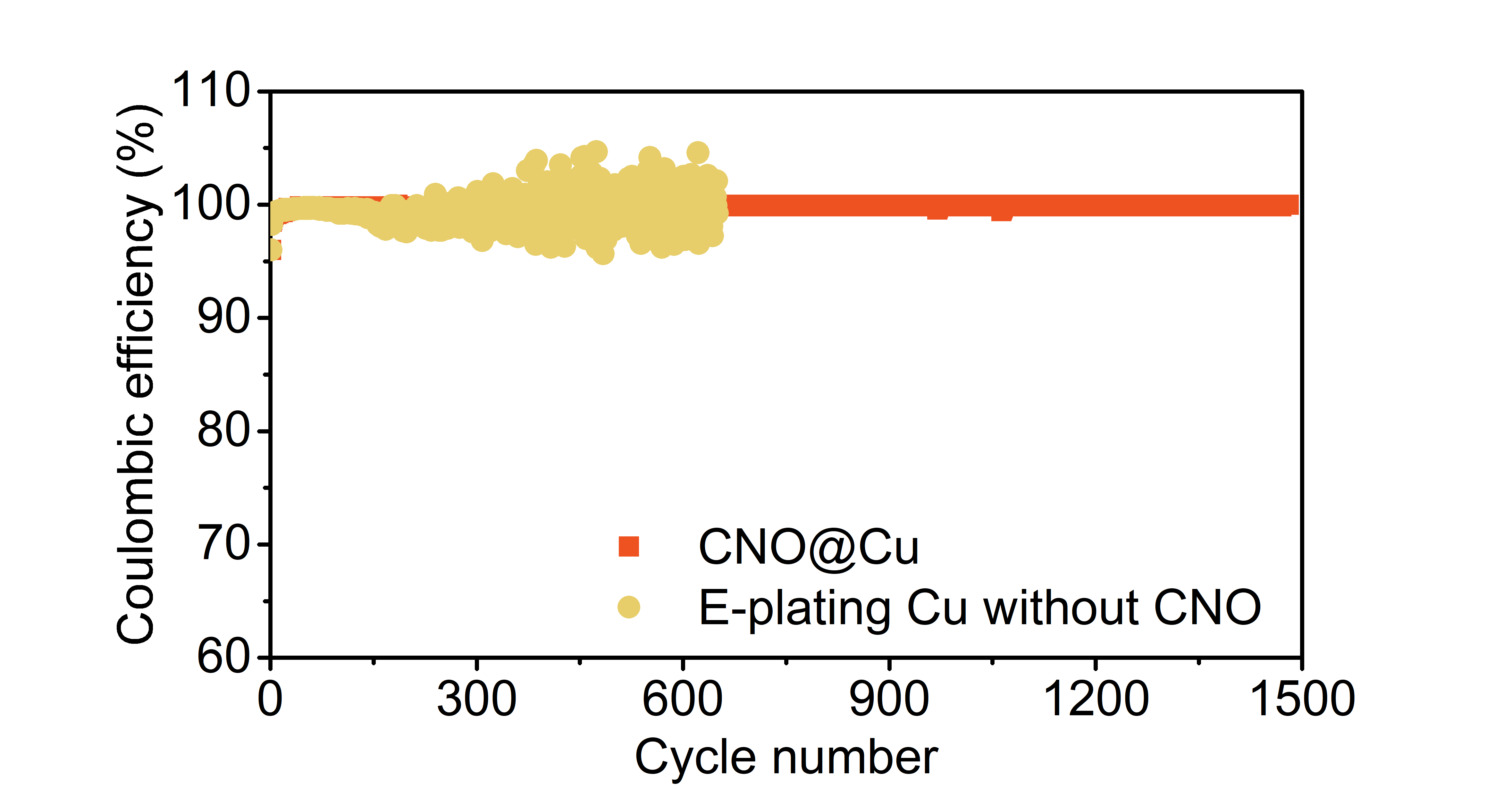


Figure S7. Asymmetric half-cell test. Cycling performance for Zn plating/stripping on electroplating Cu without CNO in the electrolyte at 5 mA/cm^2^ current density for 1 mAh/cm^2^ capacity.


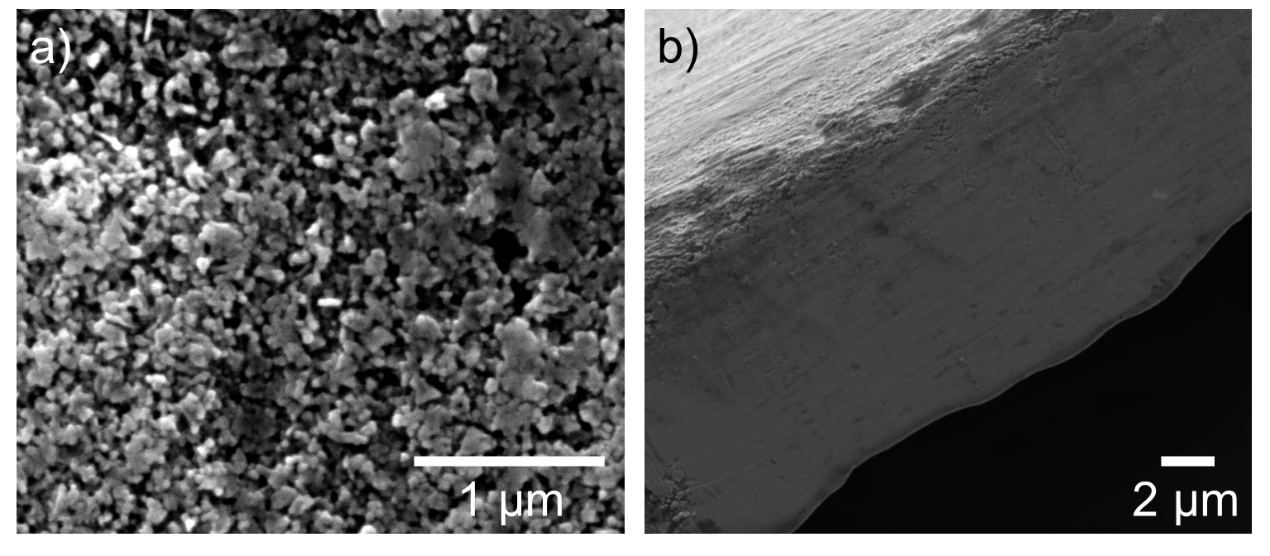


Figure S8. SEM of (a) surface and (b) cross-section of electroplating Cu without CNOs in the electrolyte


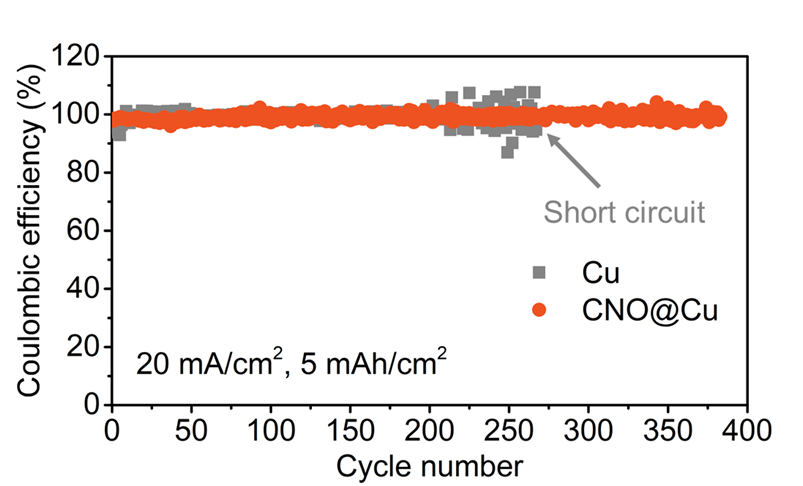


Figure S9. Cycling performance for Zn plating/stripping on different electrodes at 20 mA/cm^2^ current density for 5 mAh/cm^2^ capacity.


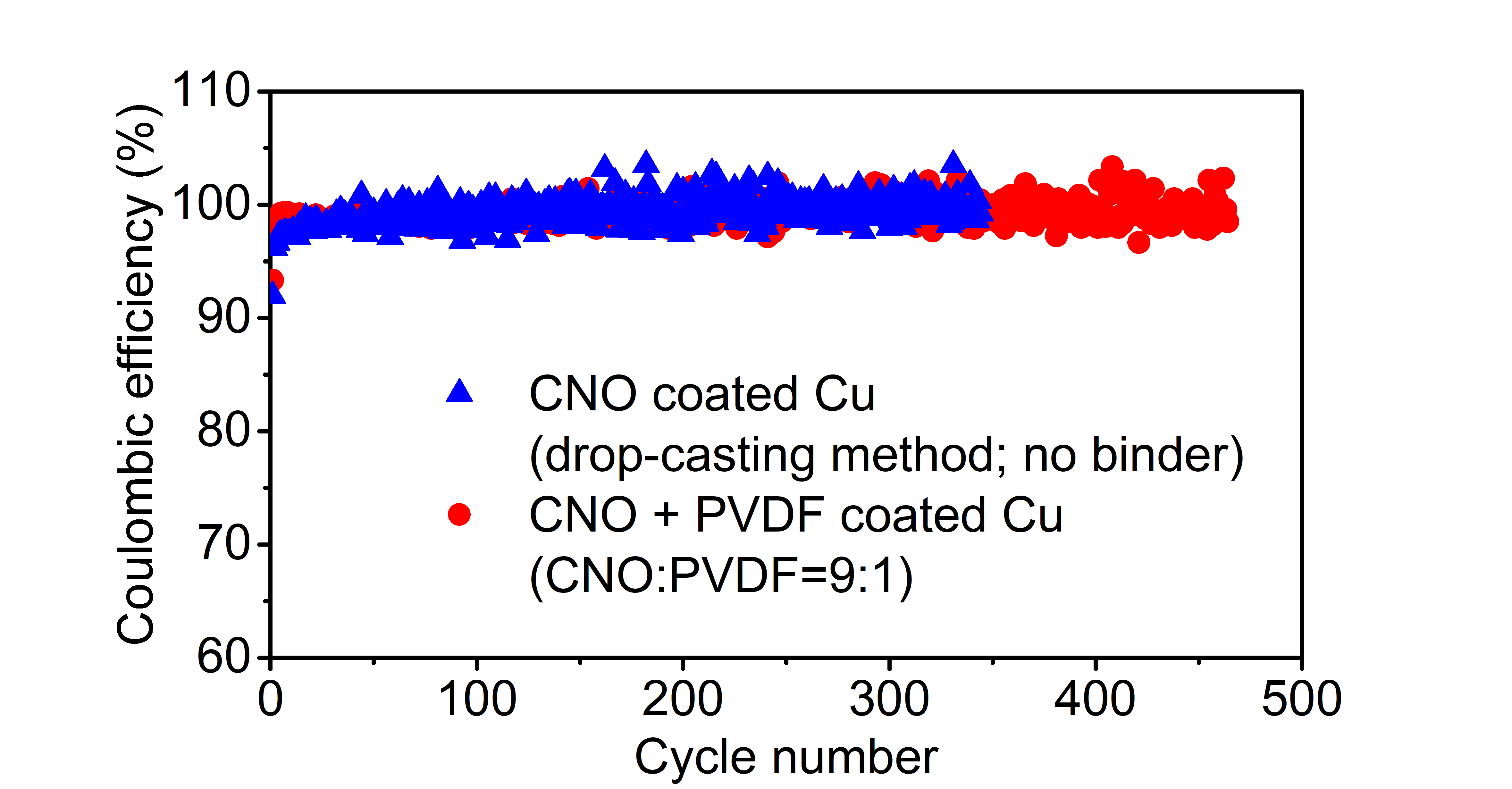


Figure S10. Asymmetric half-cell test. Cycling performance for Zn plating/stripping on different electrodes at 5 mA/cm^2^ current density for 1 mAh/cm^2^ capacity.


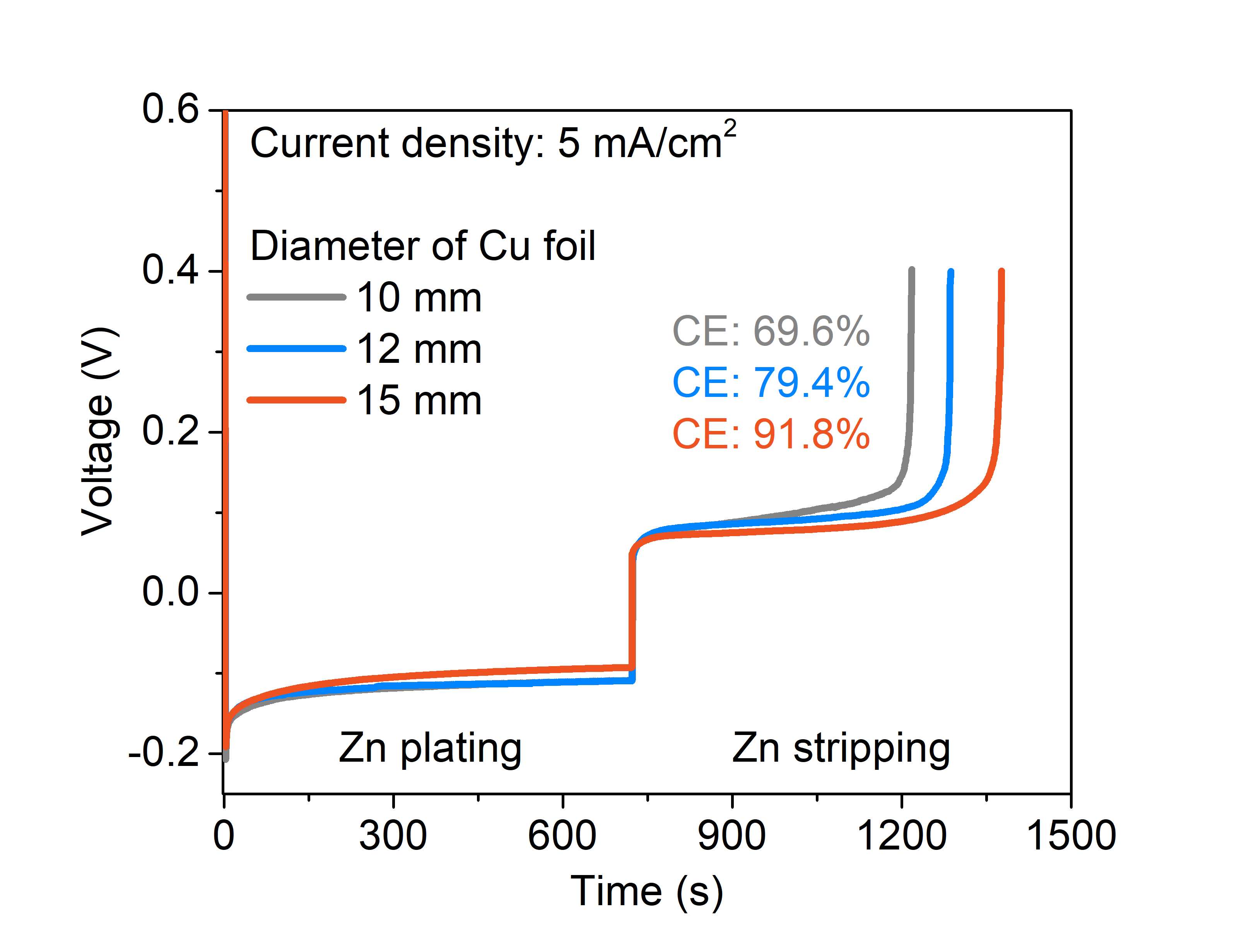


Figure S11. First-cycle voltage profiles of Cu in coin cells with different electrode sizes, showing increased CE with larger electrode area.


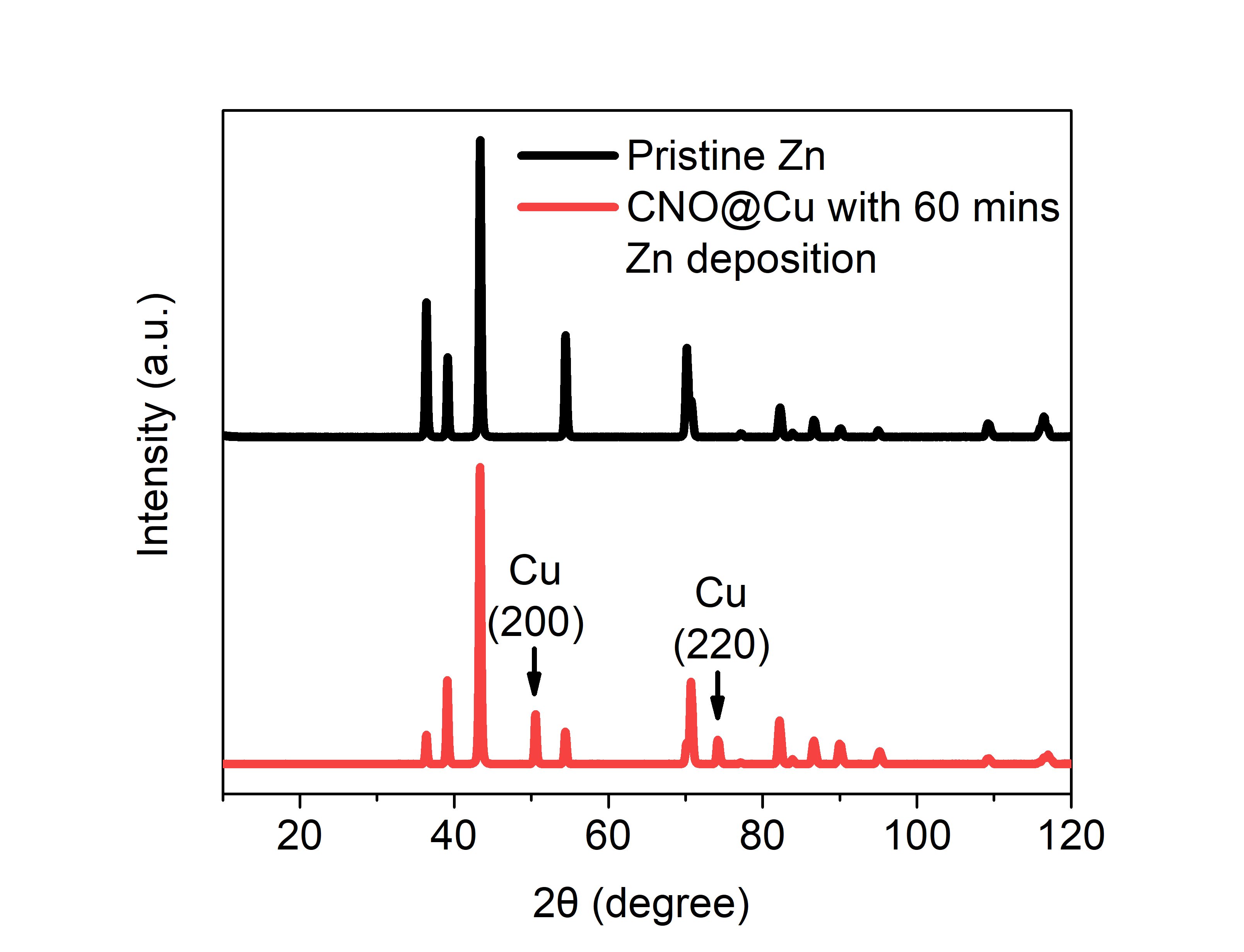


Figure S12. XRD for pristine Zn and CNO@Cu with 60 mins Zn deposition (current density: 5 mA/cm^2^).Only Cu and Zn peaks (No ZnO^2^ and ZnOH^3^ peaks) are observed in CNO@Cu with Zn position samples.


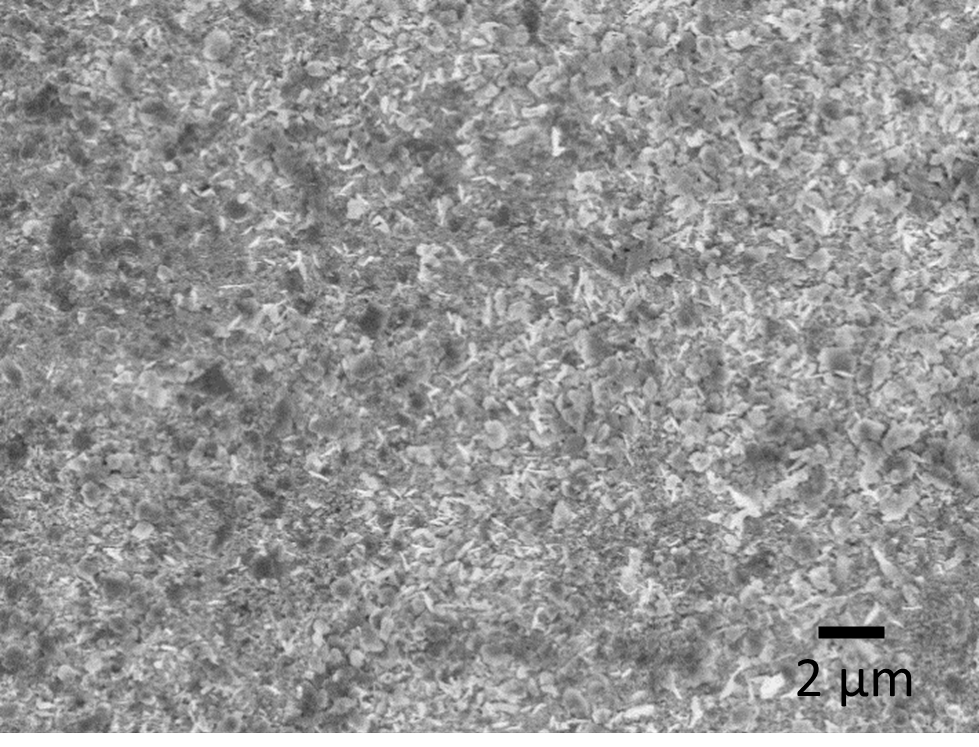


Figure S13. SEM for CNO@Cu after 100 cycles (after Zn stripping)


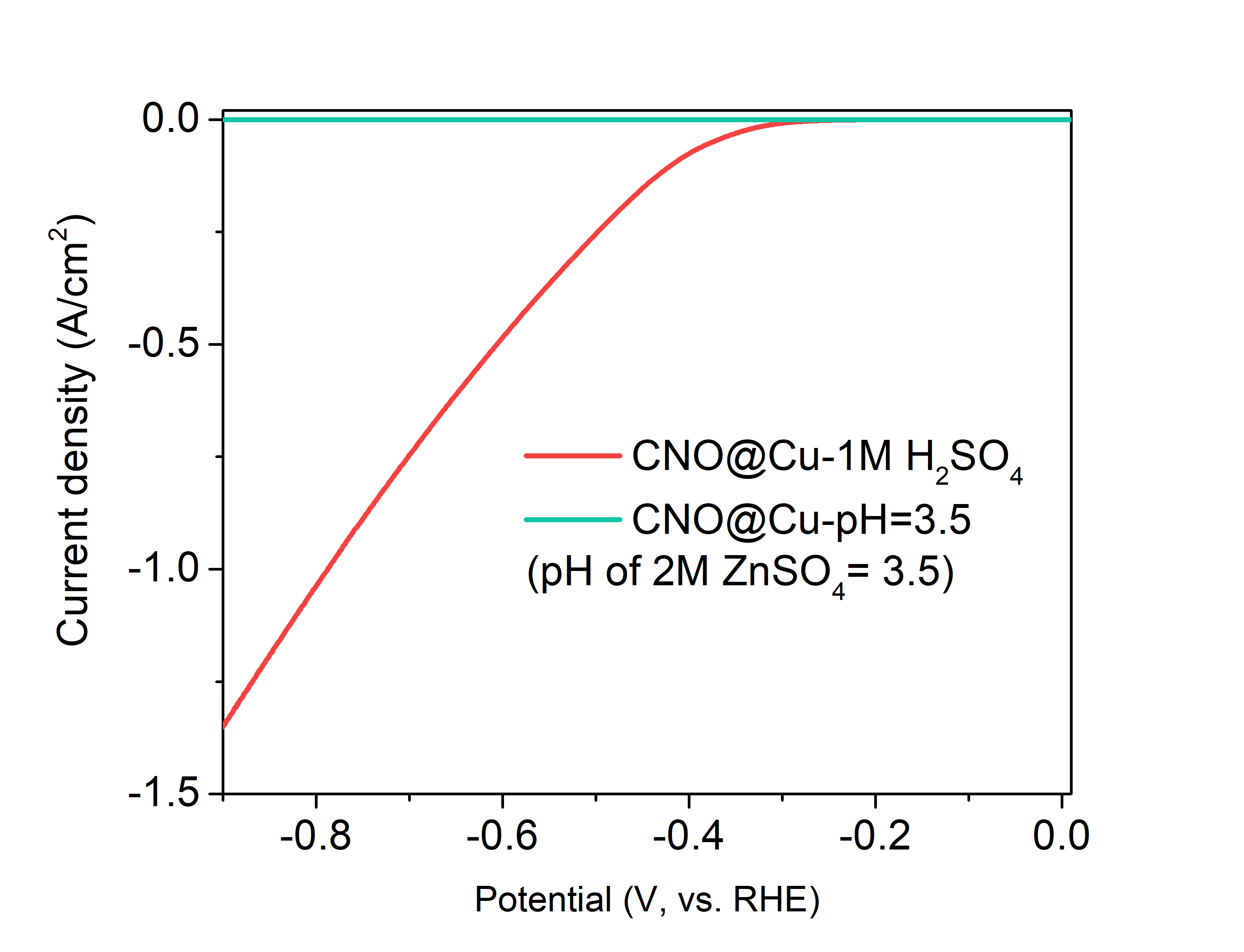


Figure S14. LSV curves of CNO@Cu electrode in 1 M H_2_SO_4_ and pH 3.5 electrolyte (no Zn^2+^) showing negligible HER activity under battery-relevant pH. Scan rate: 1 mV/s.


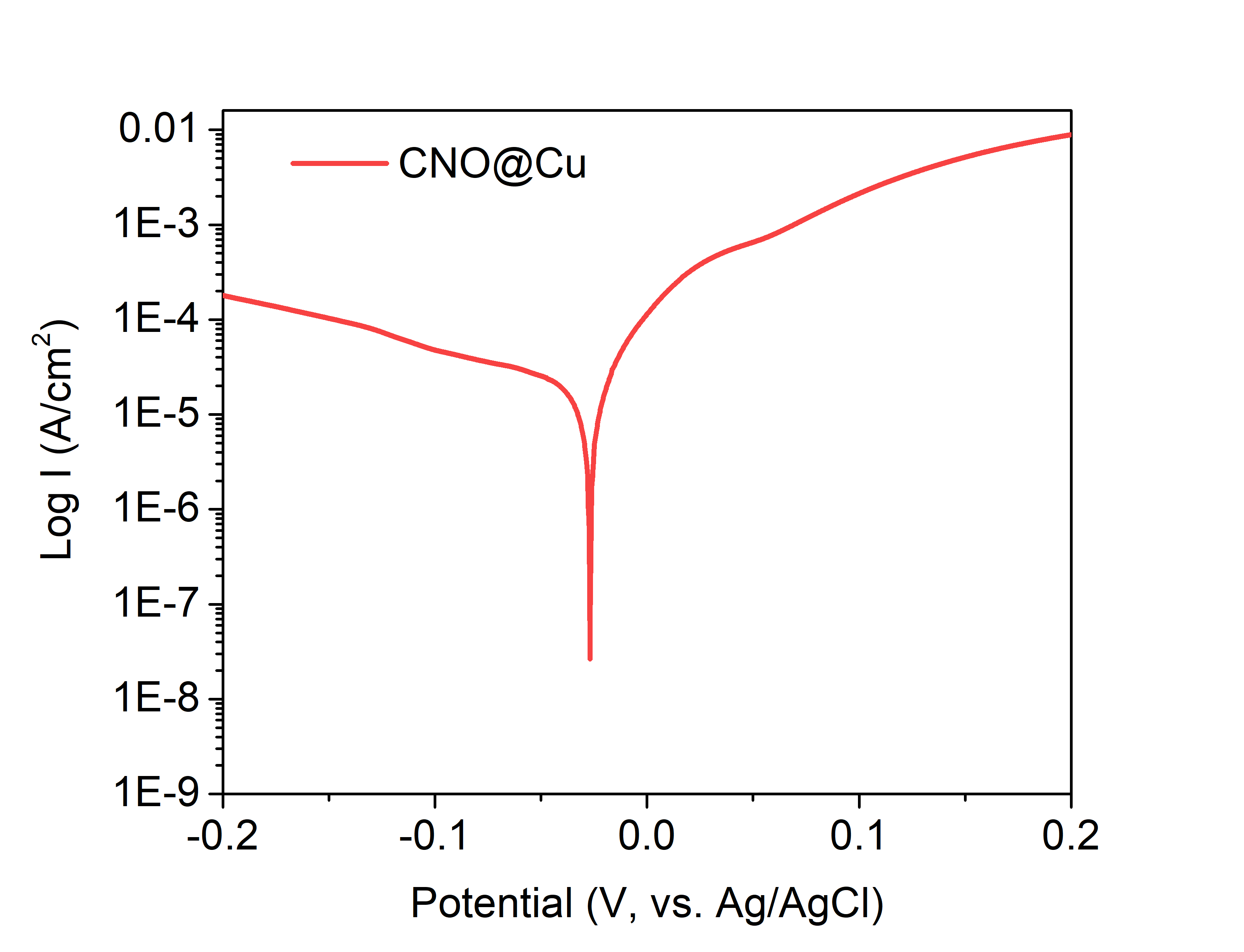


Figure S15. Tafel plot of CNO@Cu in 2 M ZnSO_4_ electrolyte, indicating low corrosion current density. Scan rate: 1 mV/s.


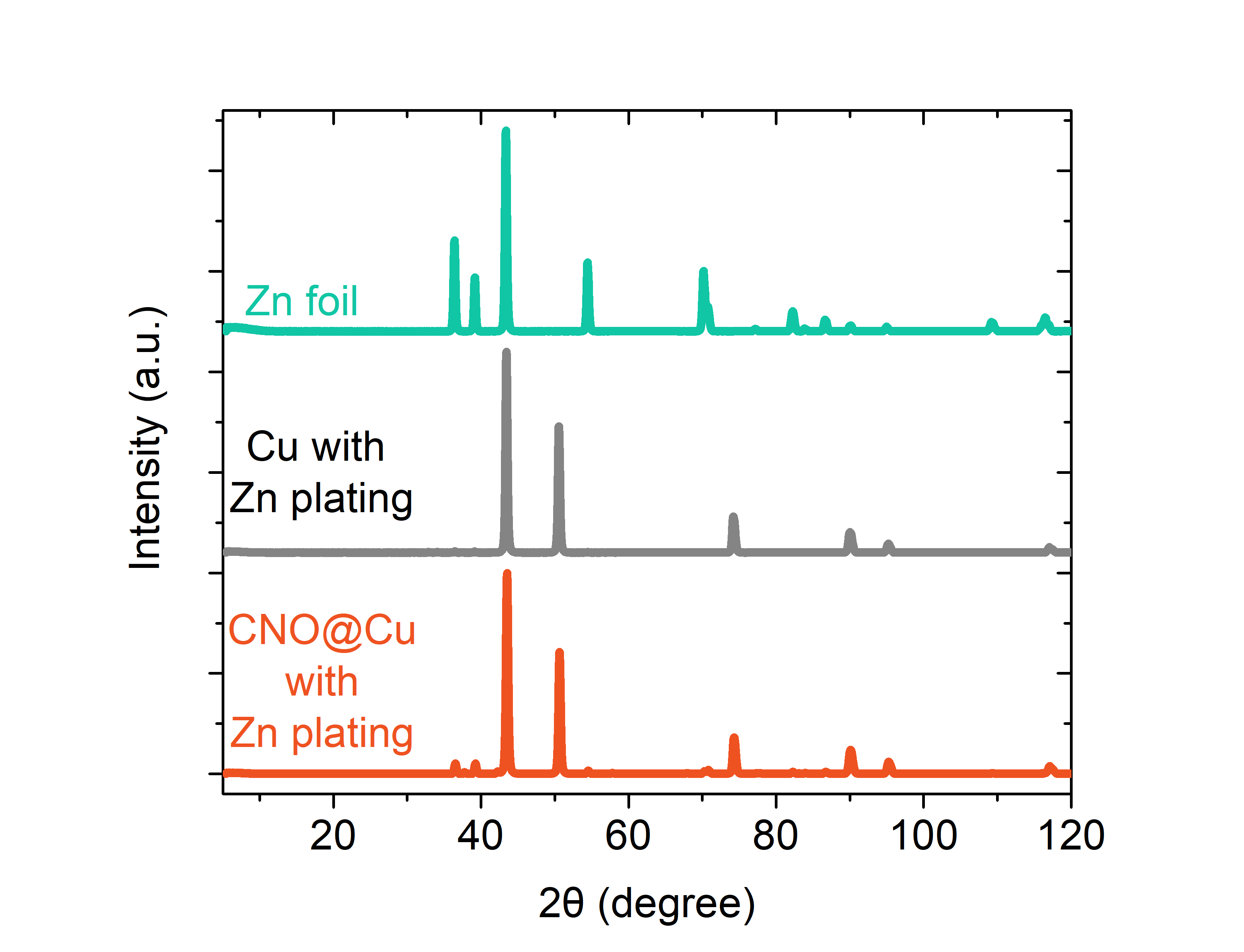


Figure S16. XRD for Zn foil, Cu with Zn plating, and CNO@Cu with Zn plating (5 mA/cm^2^ for 1 mAh/cm^2^)


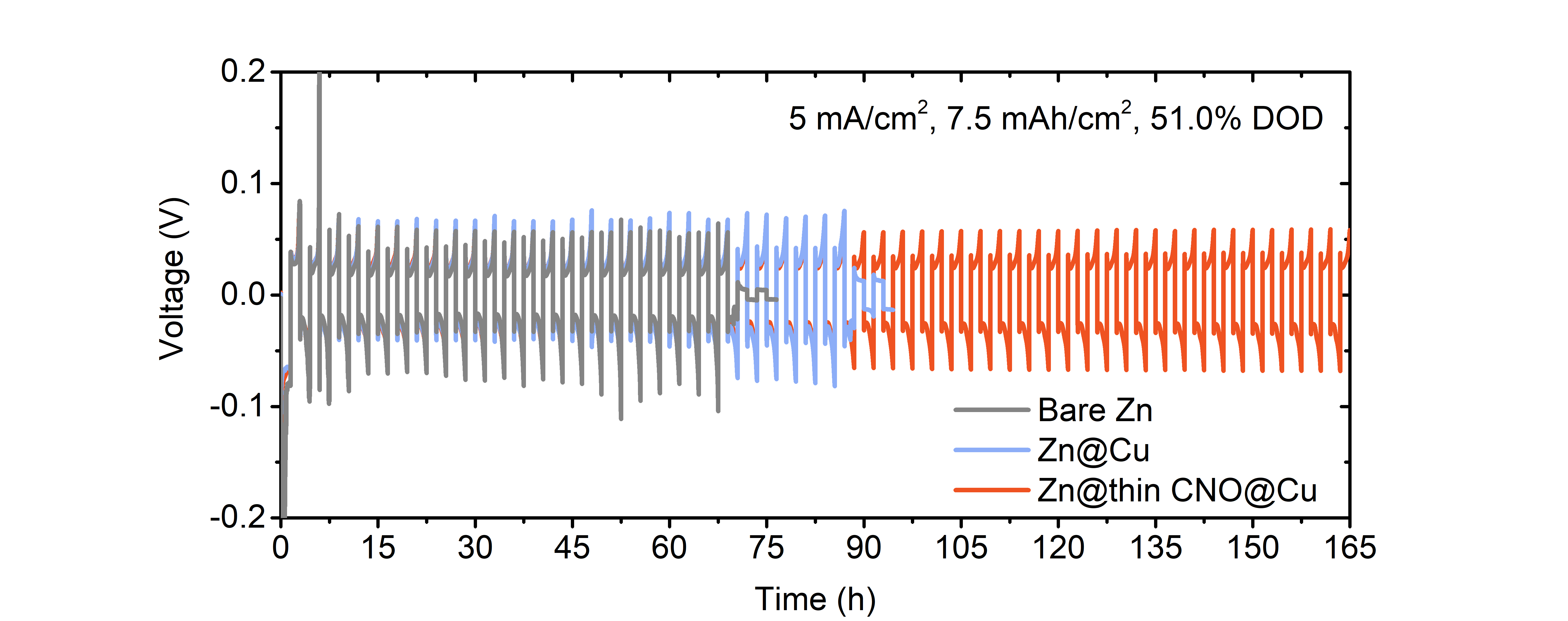


Figure S17. Symmetric cell test (thickness of Zn foil: 25 µm).


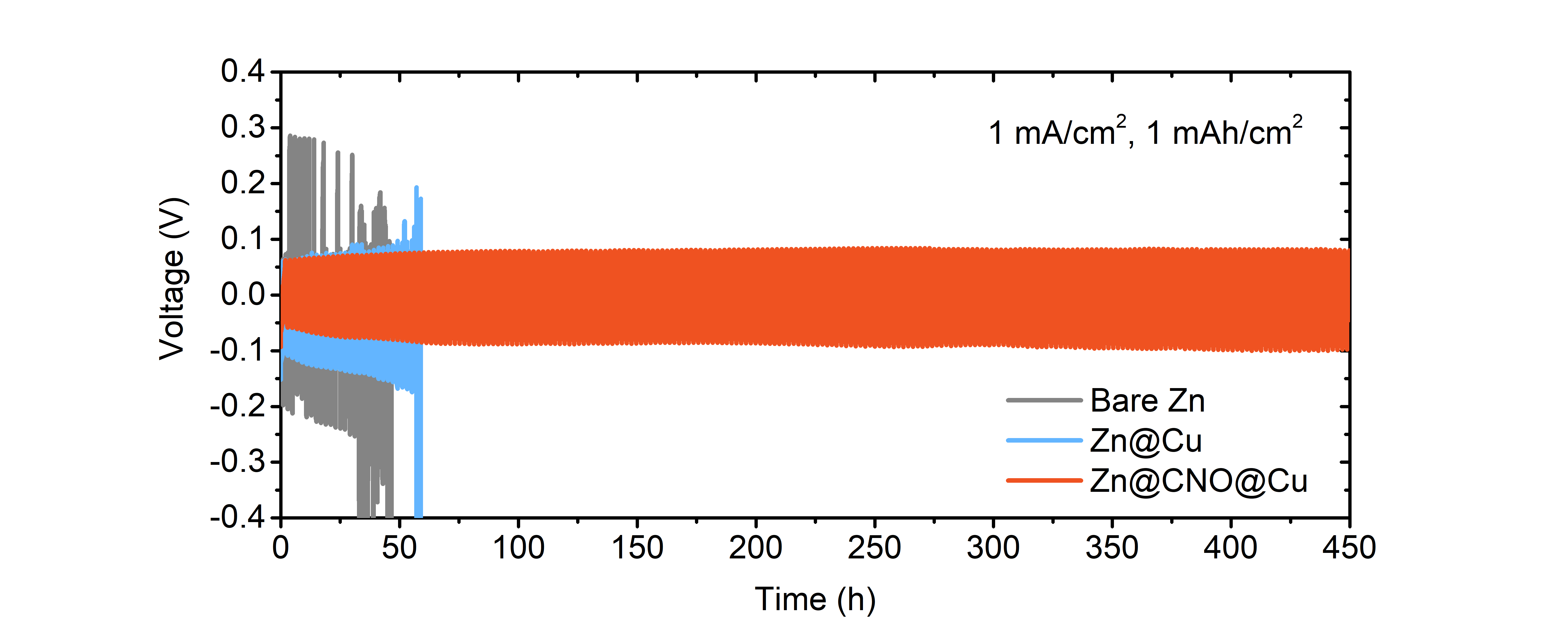


Figure S18. Symmetric cell test (thickness of Zn foil: 10 µm).


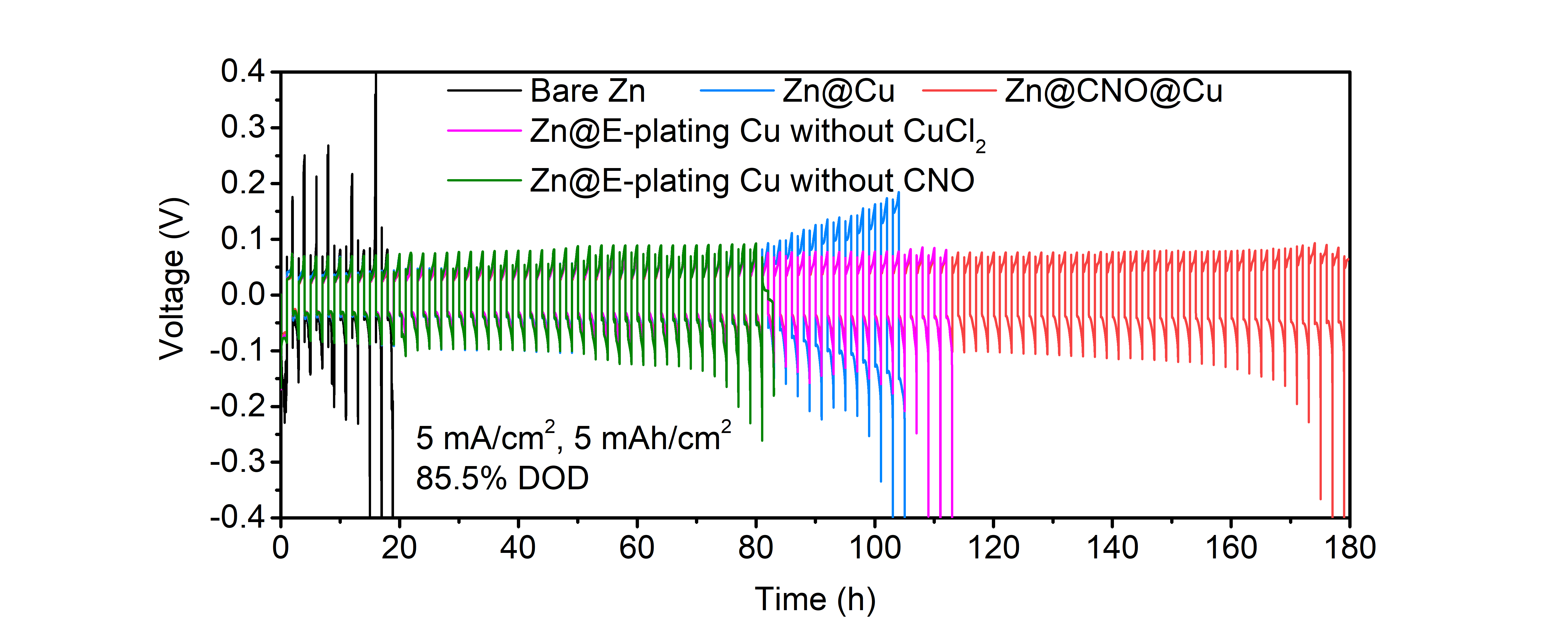


Figure S19. Symmetric cell test (thickness of Zn foil: 10 µm).


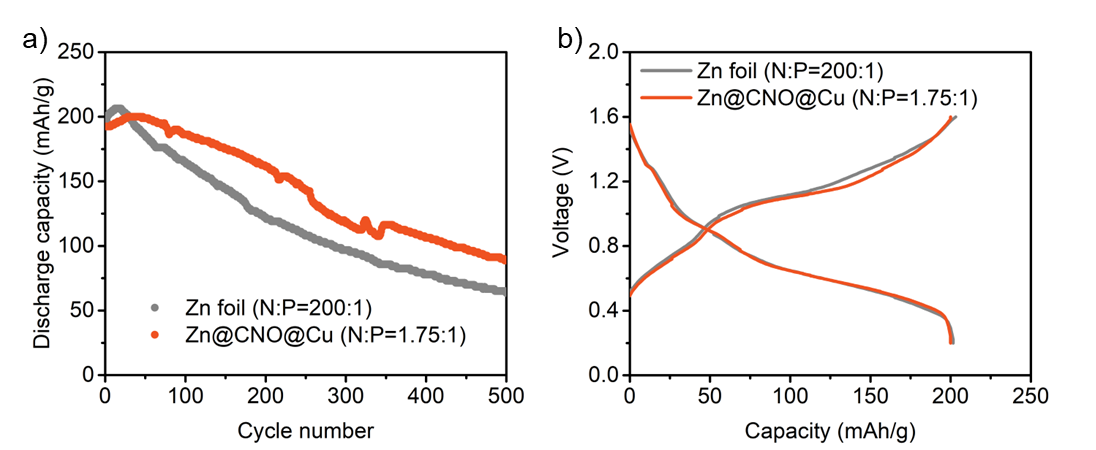


Figure S20. Full-cell test. (a) Cycling performance for different configuration ZIBs with ~1.5 mg/cm^2^ cathode material loading at 5 mA/cm^2^ current density. (b) GCD curves for different configuration ZIBs at 5 mA/cm^2^ current density.


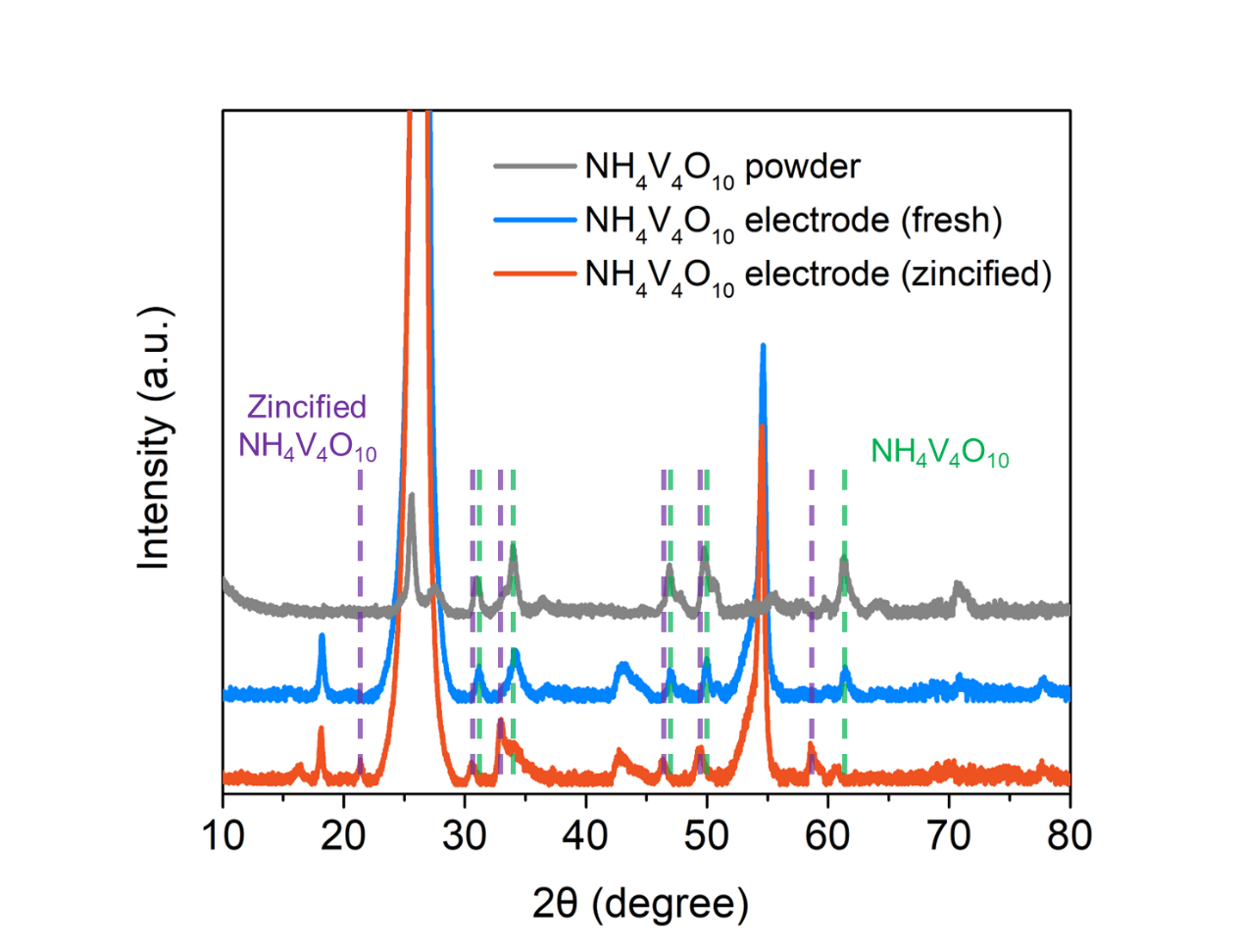


Figure S21. XRD patterns of NH_4_V_4_O_10_ powder, fresh NH_4_V_4_O_10_ electrode, and zincified NH_4_V_4_O_10_ electrode. Purple dashed lines indicate new or shifted peaks after zincification, while green dashed lines indicate peaks present in the pristine sample but absent in the zincified form.


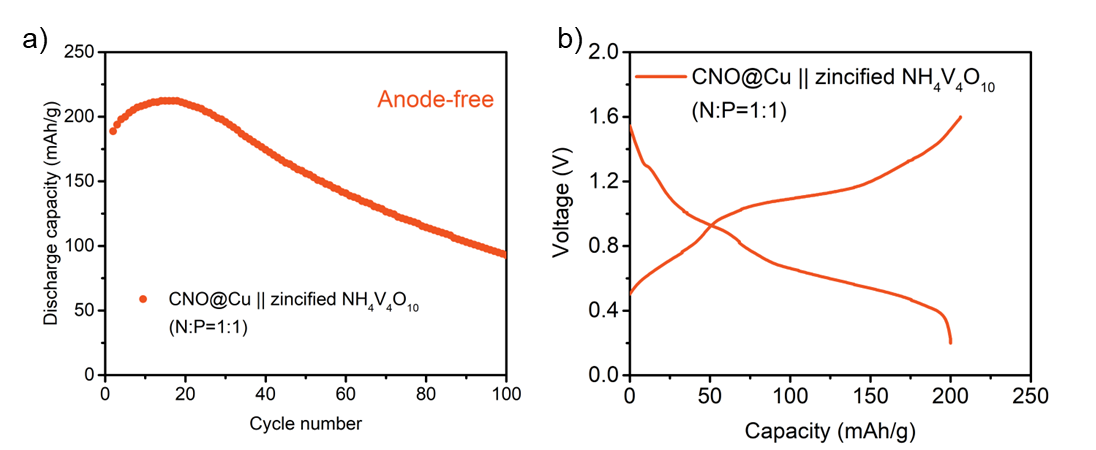


Figure S22. Full-cell test. (a) Cycling performance for different configuration ZIBs with ~1.5 mg/cm^2^ cathode material loading at 5 mA/cm^2^ current density. (b) GCD curves for different configuration ZIBs at 5 mA/cm^2^ current density.

Table S2. The volumetric energy density of the full cell (coin cell)

| Anode current collector (μm) | Anode (μm) | Separator* (μm) | Cathode (μm) |  | Cathode current collector | Volumetric energy density** (Wh/L) |
| --- | --- | --- | --- | --- | --- | --- |
| ~10 | anode free (0) | ~20 | ~70*** |  | ~10 | 194 |
| ~10 | 100 | ~20 | ~70*** |  | ~10 | 102 |

*Ref [^4^]

**Areal energy density for our full cell: 2.14 mWh/cm^2^

***Experimental measured value

**References**

1 Zhang, Y. *et al.* Hydrocarbon-Derived Graphene Nanoparticles and Their Networked Morphology. *Advanced Engineering Materials* **27**, 2402236 (2025). <https://doi.org:https://doi.org/10.1002/adem.202402236>

2 Jayswal, S. & Moirangthem, R. S. Thermal decomposition route to synthesize ZnO nanoparticles for photocatalytic application. *AIP Conference Proceedings* **2009** (2018). <https://doi.org:10.1063/1.5052092>

3 Mousavi-Kamazani, M., Zinatloo-Ajabshir, S. & Ghodrati, M. One-step sonochemical synthesis of Zn(OH)2/ZnV3O8 nanostructures as a potent material in electrochemical hydrogen storage. *Journal of Materials Science: Materials in Electronics* **31**, 17332-17338 (2020). <https://doi.org:10.1007/s10854-020-04289-4>

4 Yang, N. *et al.* Backside Coating for Stable Zn Anode with High Utilization Rate. *Advanced Materials* **36**, 2312934 (2024). <https://doi.org:https://doi.org/10.1002/adma.202312934>
